# The Master Activator of IncA/C Conjugative Plasmids Stimulates Genomic Islands and Multidrug Resistance Dissemination

**DOI:** 10.1371/journal.pgen.1004714

**Published:** 2014-10-23

**Authors:** Nicolas Carraro, Dominick Matteau, Peng Luo, Sébastien Rodrigue, Vincent Burrus

**Affiliations:** 1 Laboratory of bacterial molecular genetics, Département de biologie, Université de Sherbrooke, Sherbrooke, Canada; 2 Laboratory of microbial systems and synthetic biology, Département de biologie, Université de Sherbrooke, Sherbrooke, Canada; 3 CAS Key Laboratory of Tropical Marine Bio-resources and Ecology, South China Sea Institute of Oceanology, Chinese Academy of Sciences, Guangzhou, China; Uppsala University, Sweden

## Abstract

Dissemination of antibiotic resistance genes occurs mostly by conjugation, which mediates DNA transfer between cells in direct contact. Conjugative plasmids of the IncA/C incompatibility group have become a substantial threat due to their broad host-range, the extended spectrum of antimicrobial resistance they confer, their prevalence in enteric bacteria and their very efficient spread by conjugation. However, their biology remains largely unexplored. Using the IncA/C conjugative plasmid pVCR94ΔX as a prototype, we have investigated the regulatory circuitry that governs IncA/C plasmids dissemination and found that the transcriptional activator complex AcaCD is essential for the expression of plasmid transfer genes. Using chromatin immunoprecipitation coupled with exonuclease digestion (ChIP-exo) and RNA sequencing (RNA-seq) approaches, we have identified the sequences recognized by AcaCD and characterized the AcaCD regulon. Data mining using the DNA motif recognized by AcaCD revealed potential AcaCD-binding sites upstream of genes involved in the intracellular mobility functions (recombination directionality factor and mobilization genes) in two widespread classes of genomic islands (GIs) phylogenetically unrelated to IncA/C plasmids. The first class, SGI1, confers and propagates multidrug resistance in *Salmonella enterica* and *Proteus mirabilis*, whereas MGI*Vmi*1 in *Vibrio mimicus* belongs to a previously uncharacterized class of GIs. We have demonstrated that through expression of AcaCD, IncA/C plasmids specifically trigger the excision and mobilization of the GIs at high frequencies. This study provides new evidence of the considerable impact of IncA/C plasmids on bacterial genome plasticity through their own mobility and the mobilization of genomic islands.

## Author Summary

Multidrug resistance is a major health concern that complicates treatments of even the most common infections caused by bacteria. In recent years, IncA/C plasmids have emerged and spread in bacteria infecting humans, food-producing animals and food products, driving at the same time the dissemination of a broad spectrum of antibiotic resistance genes in environmental and in clinical settings. In this study, we have characterized the regulatory pathway that governs IncA/C plasmid dissemination. We have found that AcaCD, the master activator complex encoded by these plasmids, is not only essential for the dissemination of IncA/C plasmids but also activates unrelated mobile genetic elements in bacterial genomes, thereby further promoting the interspecies propagation of multidrug resistance and other adaptive traits at a very high frequency.

## Introduction

Multidrug resistance (MDR) is steadily increasing in Gram-negative bacteria in both community and hospital settings, and represents a growing concern worldwide [1]. MDR usually results from the adaptation of microorganisms through various mutations or from the acquisition of foreign DNA by horizontal gene transfer. In recent years, conjugative plasmids of the IncA/C incompatibility group, which are prevalent in enteric bacteria, have become a substantial threat due to their broad host-range, their extended spectrum of antimicrobial resistance and their efficient spread by conjugation [2]. IncA/C plasmids were first identified more than 40 years ago from diseased fish infected by antibiotic-resistant *Aeromonas hydrophila* and *Vibrio* spp [3], [4]. For more than three decades IncA/C plasmids received relatively little attention, but the rapid dissemination of these MDR-carrying vectors among enteric pathogens recovered from food-producing animals, food products and humans have sprung intensive research at the epidemiological and genomic level. Several IncA/C plasmids are spreading the New Delhi metallo-lactamase *bla*_NDM-1_ gene and its variants, which confer resistance to all β-lactams except for monobactams and are widely distributed throughout all Gammaproteobacteria [5]–[8]. Resistance to β-lactams, aminoglycosides, chloramphenicol, folate pathway inhibitors (sulfonamides and trimethoprim), quinolones and tetracycline is also commonly conferred by these large plasmids (ca. 140 to 200 kb) [9]–[13]. IncA/C plasmids have also been shown to mobilize in *trans* the *Salmonella* genomic island 1 (SGI1), a 43-kb chromosomal mobile element carrying a class 1 integron that confers resistance to ampicillin, chloramphenicol, streptomycin, sulfonamides and tetracycline (ACSSuT phenotype) [14]–[16]. SGI1 and related MDR-conferring genomic islands (GIs) have been found in a large variety of *Salmonella enterica* serovars and in *Proteus mirabilis* [17]. To date, the genetic regulatory network and the nature of the interactions allowing the specific mobilization of SGI1 by IncA/C helper plasmids remain largely unknown.

 Comparative genomics studies of IncA/C plasmids isolated from *Escherichia coli*, *S. enterica*, *Vibrio cholerae*, *Yersina pestis*, *Yersinia ruckeri*, *Klebsiella pneumoniae* and *Providencia stuartii* have revealed their close relationship [9], [10], [18], [19]. IncA/C plasmids share a common core set of genes exhibiting more than 99% identity that is disrupted by antibiotic-resistance conferring cassettes. While several of these conserved genes are involved in conjugative transfer (*tra* genes) and replication (*repA*), most have unknown functions. IncA/C plasmids are distantly related to the integrative and conjugative elements (ICEs) of the SXT/R391 family, which are also broadly distributed among several species of *Enterobacteriaceae* and *Vibrionaceae* [10], [19], [20]. Although many IncA/C plasmids have been fully sequenced to date, little is known about their basic biology, and most importantly about the regulation of their dissemination by conjugation.

 Previously, we have identified and characterized the IncA/C conjugative plasmid pVCR94 from the epidemic isolate *V. cholerae* O1 El Tor F1939 [19]. pVCR94 transfers at very high frequency (10^−2^ to 10^−1^) across species and genera, and mediates resistance to co-trimoxazole, chloramphenicol, streptomycin, ampicillin and tetracycline [19]. Here, we have characterized the regulatory mechanisms that control the conjugative transfer function of IncA/C plasmids. Two transcriptional repressors, *acr1* and *acr2*, were shown to repress the expression of two plasmid-encoded conserved genes, *acaC* and *acaD*, that are essential for the activation of IncA/C *tra* gene expression. Chromatin immunoprecipitation coupled to exonuclease digestion (ChIP-exo) and RNA sequencing (RNA-seq) assays were used to characterize the AcaCD regulon. Finally, bioinformatics analyses and experimental evidence revealed that the AcaCD regulon expands beyond IncA/C plasmid-borne genes to include GIs such as SGI1 and another unrelated GI of *Vibrio mimicus*. Altogether these results reveal a mechanism by which IncA/C conjugative plasmids play a role more important than previously estimated in bacterial genome dynamics.

## Results and Discussion

### Identification of repressors of IncA/C plasmids transfer

Comparative genomics previously revealed a set of six genes coding for putative transcriptional regulators in pVCR94 that are also conserved in nearly all IncA/C plasmids [19] (Table S1). To determine whether these genes are involved in the regulation of pVCR94 transfer, we constructed in-frame deletions and tested the ability of the resulting mutants to transfer by conjugation. For convenience, we carried out all of our assays using the 120,572-bp pVCR94ΔX mutant (referred to as pVCR94 in the rest of the paper), in which a single cluster containing all antibiotic resistance genes except for *sul2* conferring resistance to sulfamethoxazole has been deleted [19]. Deletion of *vcrx025*, which codes for a putative HUβ-like DNA-binding protein, had no effect on plasmid transfer (3.94×10^−3^ exconjugant/donor for pVCR94Δ*vcrx025* compared to 3.51×10^−3^ exconjugant/donor for wild-type, *P* = 0.4400, two-tailed Student's *t*-test). Despite several attempts, we were unable to delete *vcrx027*, which codes for a Cro-like transcriptional regulator, suggesting that its absence is lethal. The adjacent gene *vcrx028* is predicted to code for a toxin protein (addiction module killer protein, IPR009241), suggesting that the co-transcribed gene *vcrx027* encodes its cognate antitoxin (see Dataset S1).

**Figure 1:**
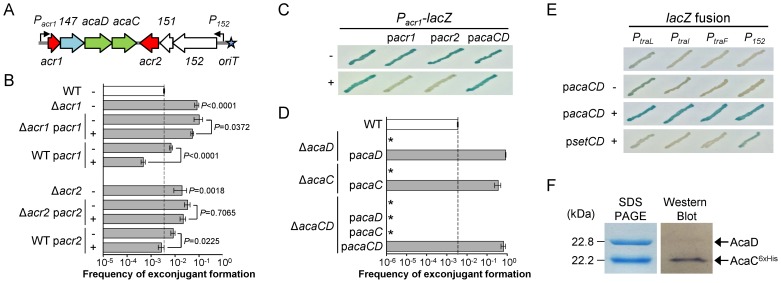
Regulation of IncA/C plasmids. (A) Schematic representation of the regulatory region of IncA/C plasmids. Genes and promoters are represented by arrowed boxes and angled arrows, respectively. Repressors and activators are shown in red and green, respectively. Genes of unknown function are shown in white and the gene coding for a putative lytic transglycosylase is shown in light blue. Locus names *vcrxXXX* are abbreviated as *XXX*. The *oriT* locus is depicted by a blue star. (B) Effect of the deletion and the overexpression of the negative transcriptional regulator-encoding genes *acr1* and *acr2*. Conjugation assays were carried out using as donors *E. coli* BW25113 Nx containing pVCR94ΔX2 (WT) or its Δ*acr1* and Δ*acr2* mutants. Complementation and overexpression assays were performed with (+) or without (−) arabinose for the expression of *acr1* (p*acr1*) or *acr2* (p*acr2*) from the inducible *P*_BAD_ promoter. *E. coli* MG1655 Rf was used as the recipient. Transfer frequencies are expressed as a number of exconjugant per recipient CFUs. The bars represent the mean and standard deviation values obtained from at least three independent experiments. Statistical analyses were performed on the logarithm value of the means using one-way ANOVA with Tukey's multiple comparison test. *P*-values are indicated next to the bars when comparison referred to WT or next to the brackets when comparing two bars. (C) The constitutive promoter of *acr1* (*P_acr1_*) is repressed by Acr1 and Acr2. Activity of *P_acr1_* was monitored from a single-copy, chromosomally integrated *lacZ* transcriptional fusion (*P_acr1_*-*lacZ*). Colorimetric assays were carried out on LB medium supplemented with 40 µg/ml of X-Gal and induction with (+) or without (−) arabinose to express *acr1*, *acr2* or *acaCD* from *P*_BAD_ on p*acr1*, p*acr2* or p*acaCD*, respectively. (D) AcaC and AcaD are essential for conjugative transfer. Transfer assays were carried out using *E. coli* BW25113 Nx containing pVCR94ΔX2 (WT) or the mutants Δ*acaC*, Δ*acaD* or Δ*acaCD*. Complementation assays were performed by expressing *acaC*, *acaD* or *acaCD* from *P*_BAD_ on p*acaC*, p*acaD* and p*acaCD*, respectively. Recipient strains and statistical analyses were as described for panel B. All *P*-values are below 0.0001 when compared to the WT. The asterisk indicates that frequency of exconjugant formation was below the detection limit (<10^−8^). (E) AcaCD is the direct activator of *tra* gene promoters. Activity of the *P_traL_*, *P_traI_*, *P_traF_* and *P_152_* was monitored from single-copy, chromosomally integrated *lacZ* transcriptional fusions. Colorimetric assays were performed as described in panel C with expression of *acaCD or setCD* from *P*_BAD_ on p*acaCD* or p*setCD* (pGG2B), respectively. (F) AcaD co-purifies with AcaC. Coomassie blue-stained SDS-PAGE and Western blot analysis of AcaC purified using a Ni-NTA affinity chromatography. AcaD and 6×His-tagged AcaC were co-expressed in *E. coli* BL21(DE3) from p*acaDC* ^6×His^. Western blot analysis was performed using a monoclonal antibody against the 6×His-tag.

The remaining four putative regulator genes cluster near the origin of transfer (*oriT*) (Figure 1A). Deletion of either *vcrx146* (*acr1*), which codes for a putative Ner-like DNA-binding protein, or *vcrx150* (*acr2*), which codes for a predicted H-NS-like DNA-binding protein, resulted in a 23- and 5.6-fold increase of the frequency of transfer, respectively, thereby suggesting that both genes code for repressors of pVCR94 transfer (Figure 1B). For this reason, *vcrx146* and *vcrx150* were renamed *acr1* and *acr2*, for IncA/C repressors 1 and 2, respectively. Complementation of the Δ*acr1* and Δ*acr2* mutations by ectopic expression of the corresponding genes under control of an arabinose-inducible promoter (*P*_BAD_) did not restore the wild-type transfer frequency (Figure 1B). However, overexpression of either *acr1* or *acr2* decreased transfer of wild-type pVCR94 by 14 and 3 fold, respectively. Since *acr1* is the first gene of an operon structure (see Dataset S1) containing the putative transcriptional activator genes *vcrx148* (*acaD*) and *vcrx149* (*acaC*) (see below), we tested whether *acr1* or *acr2* are able to repress expression from *P_acr1_*, the promoter driving expression of the operon containing *acr1* (Figure 1A). To do this, we cloned *P_acr1_* upstream of a promoterless *lacZ* gene and monitored the β-galactosidase activity upon expression of *acr1* or *acr2* driven by *P*_BAD_. While *P_acr1_* was transcriptionally active in the absence of arabinose, no β-galactosidase activity was detected upon expression of either *acr1* or *acr2*, confirming that both proteins are capable of directly repressing expression from *P_acr1_* (Figure 1C).

### AcaC and AcaD are key activators of IncA/C plasmids transfer

**Figure 2:**
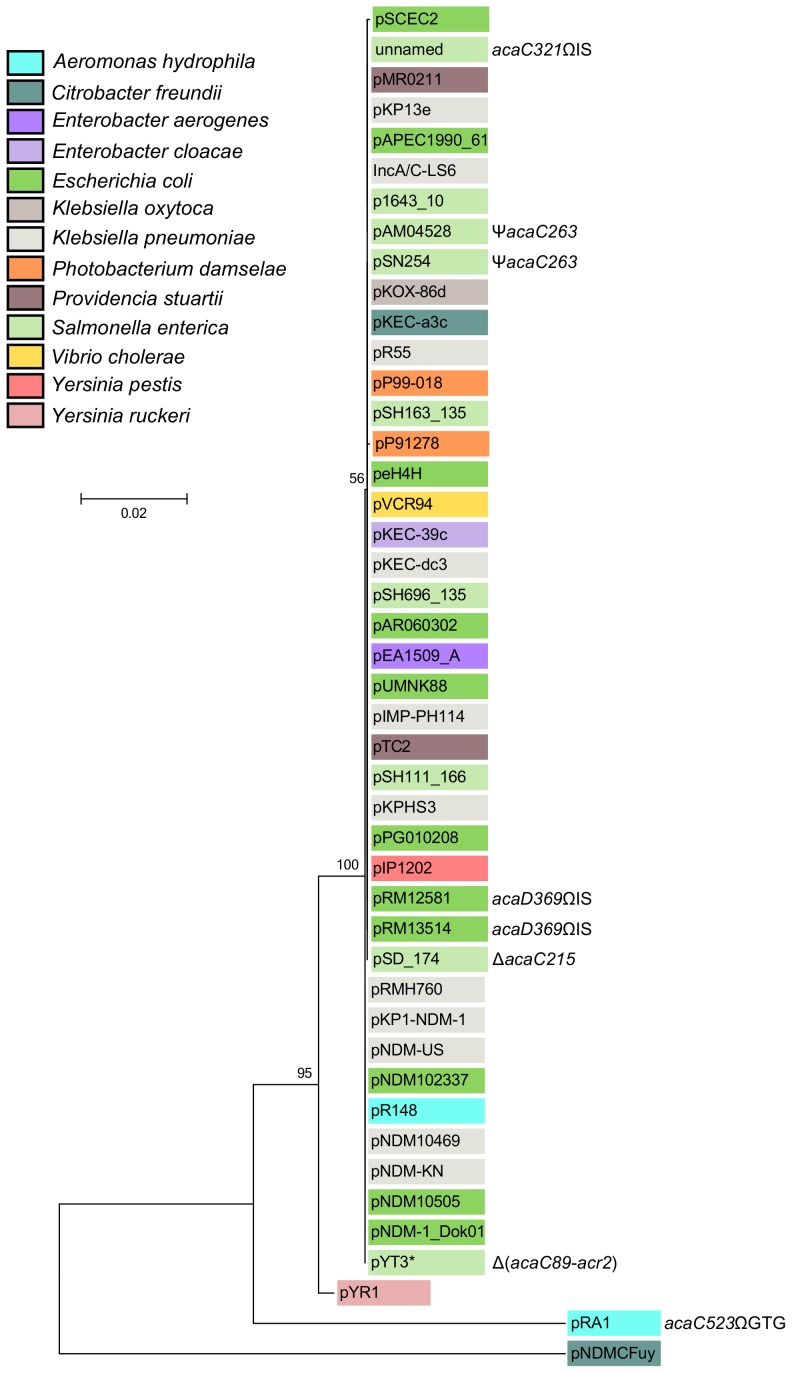
Molecular phylogenetic analysis of the *acr1*-*vcr147*-*acaDC*-*acr2* locus by Maximum Likelihood method. The evolutionary history was inferred by using the Maximum Likelihood method based on the Kimura 2-parameter model [61]. The tree with the highest log likelihood (−5461.6977) is shown. The percentage of trees in which the associated taxa clustered together is shown next to the branches. A discrete Gamma distribution was used to model evolutionary rate differences among sites (5 categories (+G, parameter = 0.6567)). The tree is drawn to scale, with branch lengths measured in the number of substitutions per site. The analysis involved 45 nucleotide sequences (Table S2). Codon positions included were 1st+2nd+3rd+Noncoding. There were a total of 2469 positions in the final dataset. Evolutionary analyses were conducted in MEGA6 [58]. The background color of each leaf indicates the original host species from which each plasmid was isolated. Insertions (Ω), deletion (Δ) and frameshift (Ψ) mutations are indicated where appropriate. *, Although pYT3 shares sequence identity with the IncA/C plasmids, it lacks an IncA/C-specific replication initiation gene (*repA*) and contains an IncFIB replicon instead (Figure S1) [62].

The cluster of genes *acr1*-*vcrx147*-*acaDC*-*acr2* is extremely well conserved and remain syntenic among all IncA/C plasmids except for XCN1_p from *Xenorhabdus nematophila* in which it is completely absent (Figures 2 and S1). Interestingly, the IncA/C plasmids pAM04528 and pSN254 from *S. enterica*, which were reported to be non-self-transferable [10], [21], [22], code for a truncated AcaC protein resulting from a frameshift mutation (*acaC263*) (Figure 2), suggesting a key role of *acaC* in the activation of IncA/C-plasmid transfer. Unfortunately, no data is currently available about the intercellular mobility of five other plasmids coding for truncated AcaC or AcaD proteins (Figure 2) [23], [24]. However, pRA1 was reported to transfer at a frequency of 10^−3^ [9] despite a GTG insertion at the 3′ end of *acaC* (*acaC523*ΩGTG) that slightly alters the primary sequence of AcaC C-terminus (Figure 2).

Pfam analyses (database v27.0) revealed that the conserved gene products AcaC and AcaD exhibit weak homology with the FlhC (Pfam PF05280) and FlhD (PF05247) domains, respectively (Table S1) [19]. FlhC and FlhD form a heteromeric complex playing a key role in the transcriptional activation of flagellar genes in Gram-negative bacteria such as *E. coli* [25]. We thus hypothesized that AcaC and AcaD (IncA/C activator subunits C and D) could acts as the master activator of *tra* genes in IncA/C plasmids. We constructed three null mutants, Δ*acaC*, Δ*acaD* and Δ*acaCD*, of pVCR94 and tested their ability to transfer by conjugation. All three mutations abolished conjugative transfer, thereby confirming that *acaCD* plays an essential role for IncA/C plasmid transmission (Figure 1D). *Trans*-complementation of these mutations under control of *P*_BAD_ restored and even outperformed transfer of pVCR94 (Figure 1D). Importantly, the Δ*acaCD* mutant could not be complemented by expression of either *acaC* or *acaD*. Only the simultaneous expression of both *acaC* and *acaD* restored the transfer of pVCR94Δ*acaCD*, strongly suggesting that these two genes code for an FlhCD-like activating complex (Figure 1D). AcaC and AcaD share 34% and 23% identity with the master activator subunits SetC and SetD encoded by SXT/R391 ICEs. As expected from the very poor percentage of identity, the Δ*acaCD* mutation was not complemented by *setCD* expressed from the IPTG-inducible promoter *P_tac_* (Figure S2).

To test whether AcaCD is necessary and sufficient to drive the expression of *tra* genes of pVCR94, we cloned the promoter regions of *traL* (*P_traL_*), *traI* (*P_traI_*) and *traF* (*P_traF_*) upstream of a promoterless *lacZ* gene. The genes *traL*, *traI* and *traF* are likely candidate for AcaCD activation as *traI* codes for the predicted relaxase that would initiate conjugative transfer of pVCR94 at *oriT* while *traL* and *traF* code for two predicted sex pilus assembly proteins. We also tested the promoters *P_acr1_* and *P_152_* that drive the expression of *acaCD* and *vcrx152*, respectively (Figure 1A). All but *P_acr1_* were inactive in the absence of *acaCD*, without arabinose induction or upon expression of *setCD* (Figure 1C, E). The constitutive expression from *P_acr1_* seemed to remain unaffected by AcaCD overexpression. In contrast, expression of AcaCD alone in cells lacking pVCR94 was sufficient to trigger the expression from *P_traL_*, *P_traI_*, *P_traF_* and *P_152_* (Figure 1E).

Collectively, these results are consistent with a model in which *acr1* represses *acaCD* expression and its own, thereby preventing expression of the *tra* genes. Accordingly, under the appropriate conditions, repression by Acr1 would be alleviated, allowing expression of *acaCD* that in turn would enable expression of the *tra* genes and other genes such as *vcrx152*. Although *acr2* seems to be part of the same operon-like structure as *vcrx152*, its expression is not up-regulated by AcaCD and likely not driven by *P_152_* (Dataset S1). Instead *acr2* seems to be constitutively expressed. As a mild H-NS-like general repressor, Acr2 could dampen *acr1* and *acaCD* expression to prevent overexpression of the conjugative machinery, which is likely deleterious to the fitness of the host [26]. The H-NS-like protein Sfh encoded by the IncHI1 conjugative plasmid R27 has been shown to provide a stealth function helping the transmission of R27 into a naïve host by preventing titration of the cellular pool of H-NS by the A+T-rich sequences of the plasmid [27]. The locus occupied by *acr2* in IncA/C plasmids contains the gene *int* in the SXT/R391 ICEs (Figure S3) [20]. *int* codes for the integrase which allows SXT/R391 ICEs to remain quiescent in the host chromosome [28].

Although the regulation loci of IncA/C conjugative plasmids and SXT/R391 ICEs encode similar transcriptional activators (AcaCD and SetCD, respectively), IncA/C plasmids carry *acr1* and an extra *acr2* gene, while lacking a homolog of *setR* (Figure S3). In SXT/R391 ICEs, *setR* codes for a λ CI-related transcriptional repressor that prevents the expression of *setC* and *setD* [29], [30]. Like λ CI, SetR responds to DNA damage by RecA*-dependent autoproteolysis, which alleviates the repression of *setCD*, thereby allowing excision and transfer of SXT/R391 ICEs [30]. Consistent with the absence of a *setR* homolog, transfer of IncA/C plasmids has been shown to be *recA*-independent [19].

### AcaC and AcaD assemble as a heteromeric activator complex

Because deletion of either *acaC* or *acaD* abolished pVCR94 transfer, and both mutations could be individually complemented in *trans*, we sought to test whether AcaC and AcaD could form a heteromeric transcriptional activator complex. To investigate this possibility, we constructed a C-terminally 6×His-tagged version of *acaC*, *acaC* ^6×His^ that was expressed together with *acaD* from *P_tac_*. AcaC^6×His^ was purified by Ni-NTA affinity chromatography and the sample was analyzed on a 12% SDS-polyacrylamide gel. Interestingly, two bands were detected after Coomassie blue staining with molecular weights consistent with AcaC and AcaD (Figure 1F). Western blot assays using anti-6×His-tag antibodies only revealed the smallest 22.2-kDa band which corresponds to AcaC^6×His^. The additional band that co-purified with AcaC^6×His^ has a molecular weight of 22.8 kDa and was confirmed to be AcaD by mass spectrometry. Therefore, our results suggest that, like FlhC and FlhD, AcaC and AcaD assemble as a heteromeric complex.

### Identification of AcaCD targets in pVCR94

To get a better understanding of the mechanisms governing transfer regulation of IncA/C conjugative plasmids, and identify genes of pVCR94 expressed under the direct control of AcaCD, we undertook an exhaustive characterization of the AcaCD regulon using transcriptome sequencing and ChIP-exo [31]. For these experiments, we used *E. coli* MG1655 Rf carrying pVCR94Δ*acaCD* and the same strain bearing a chromosomally integrated single copy of p*acaDC* ^3×FLAG^ that expresses the native AcaD subunit along with a C-terminal 3×FLAG tagged AcaC subunit under control of *P_tac_*. Based on the transfer frequency of the Δ*acaCD* mutant complemented with *acaDC* ^3×FLAG^, we concluded that the 3×FLAG did not significantly affect the function of the tagged AcaC subunit compared to its wild-type counterpart (Figure S2).

**Figure 3:**
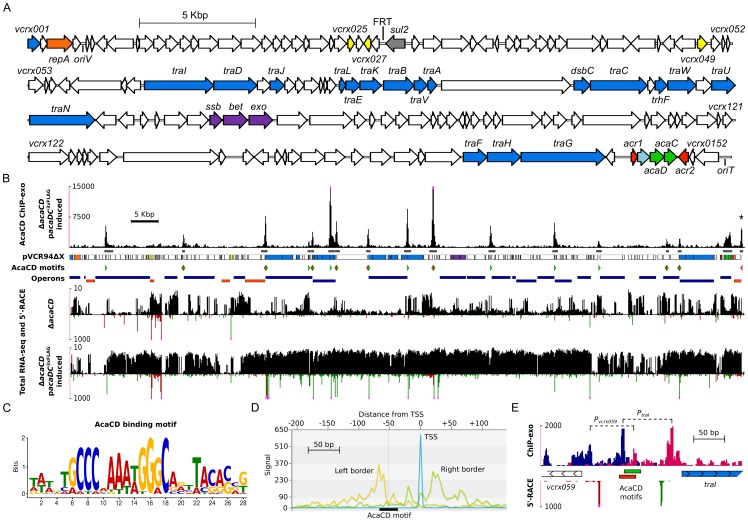
In-depth analysis of the AcaCD regulon in pVCR94. (A) Genetic organization of pVCR94ΔX adapted from Carraro *et al.* [19]. The circular map of the plasmid was linearized at the start position of gene *vcrx001*. The locations and orientations of ORFs are indicated by arrowed boxes. Colors are coded by function as follow: white, unknown; blue, conjugative transfer; light blue, lytic transglycosylase; orange, replication; gray, antibiotic resistance; yellow, putative regulatory function; purple, recombination; green, activator; red, repressor. The origin of replication (*oriV*) and the origin of transfer (*oriT*) are also indicated. The position of the scar resulting from the deletion of the antibiotic resistance gene cluster is indicated by FRT. *sul2*, resistance to sulfamethoxazole. (B) Results of ChIP-exo and RNA-seq experiments on *E. coli* MG1655 Rf carrying pVCR94Δ*acaCD* with or without a single chromosomal copy of p*acaDC* ^3×FLAG^ expressing the native AcaD subunit along with a C-terminal 3×FLAG-tagged AcaC subunit induced by IPTG. The first track plots the number of ChIP-exo reads mapped as a function of the position in pVCR94ΔX (black bars). Pink dots at the top of peaks indicate a signal beyond the represented y-axis maximal value. The second track shows the position of ChIP-exo enrichment peaks found by MACS [63] (dark gray). The asterisk at the top of the rightmost peak indicates a MACS false negative result manually incorporated in the figure. The third track is a representation of the pVCR94ΔX genes using the same color code as in panel A. The fourth track indicates the position of the AcaCD-binding motifs found by MAST [64] within ChIP-exo peaks using the AcaCD logo shown in panel C. Green arrows, motifs on positive DNA strand; red arrows, motifs on negative DNA strand. The fifth track represents Rockhopper's [65] predicted operons. Dark blue, operons transcribed from positive DNA strand; orange, operons transcribed from negative DNA strand. The remaining four tracks show the total RNA-seq read densities (black bars; log scale) and the genome-wide 5′-RACE signals (green and red bars respectively on the positive and negative DNA strands; linear scale) for cells harboring either pVCR94Δ*acaCD* or pVCR94Δ*acaCD* complemented with p*acaDC* ^3×FLAG^ and induced by IPTG. Pink dots at the top of 5′-RACE signals indicate a signal beyond the represented y-axis maximal value. (C) Motif sequence recognized by AcaCD in pVCR94ΔX obtained by MEME [64] using the AcaCD-binding sequences generated from ChIP-exo experiments. (D) VAP aggregate profile [66] showing ChIP-exo and 5′-RACE density signals centered on the AcaCD-binding motif (black box). Yellow line, density of reads mapping on the positive DNA strand (Left border); green line, density of reads mapping on the negative DNA strand (Right border). X-axis displays the distance in nucleotides from the aggregated transcription start site shown in blue (TSS). (E) Organization of *vcrx059* and *traI* divergent promoters revealed by ChIP-exo and 5′-RACE for pVCR94Δ*acaCD* complemented with IPTG-induced p*acaDC* ^3×FLAG^. The first track plots ChIP-exo read densities at single nucleotide resolution as in panel D. Dark blue, density of reads mapping on the positive DNA strand; magenta, density of reads mapping on the negative DNA strand. The second track shows the two AcaCD-binding motifs found by MAST within the ChIP-exo peak between *vcrx059* (white arrows) and *traI* (blue arrows) genes. Motif corresponding to the positive DNA strand is represented in green and motif corresponding to the negative DNA strand is shown in red. The last track represents 5′-RACE signals as described in panel B. The exonuclease-protected regions of the *vcrx059* and *traI* promoters are indicated by dashed lines.

ChIP-exo data analyses revealed 17 major AcaCD enrichment peaks, all located within intergenic regions (Figure 3A, B and Table S3). Most of the genes or operons found downstream of these peaks play key roles in conjugative transfer (Figure 3, Figure S4 and Table S3). For instance, AcaCD-binding sites were found upstream of the *traL*, *traV*, *dsbC*, *traN* and *traF* genes that are predicted to be involved in the formation of the mating pore. One peak was also found upstream of the gene coding for the putative relaxase gene *traI*. The presence of an AcaCD-binding site is also well correlated with transcriptional activity since the expression of 86 out of 152 genes, including genes located downstream of AcaCD-binding sites, is clearly increased under the same conditions used for ChIP-exo when compared to a Δ*acaCD* mutant (Figure 3B and Dataset S1). In contrast, the vast majority of genes that are not significantly affected by the expression of AcaCD appeared to be either inactive or likely constitutively expressed. Their function is unknown or not directly tied to conjugative transfer. Examples of such genes in pVCR94 include *repA* (*vcrx003*) involved in plasmid replication, a putative toxin-antitoxin system (*vcrx028* and *vcrx027*), the *sul2* (*vcrx029*) gene conferring resistance to sulfamethoxazole, as well as *acr1* and *acr2* that negatively regulate the expression of the *acr1*-*vcrx147*-*acaDC* operon (Figure 1A, C and Dataset S1).

*De novo* motif discovery of DNA sequences bound by AcaCD was carried out using MEME (Multiple Em for Motif Elicitation) [32] (Figure 3C). We next used MAST (Motif Alignment and Search Tool) [33] to determine the precise location of potential AcaCD-binding motifs on the entire sequence of pVCR94 (Figure 3B, Figure S4, and Table S3). Statistically significant motifs that were localized within ChIP-exo peaks were conserved. We next analyzed the localization of AcaCD-binding motifs relative to transcription start sites obtained using a genome-wide 5′-RACE (Rapid Amplification of cDNA Ends) methodology (Figure 3B, D, Figure S4 and Table S3). We observed a similar promoter profile, compatible with a class 2 activator [34], for all transcription start sites located between an AcaCD-binding motif and a gene in the same orientation (Figure S4). This interpretation is consistent with our high resolution ChIP-exo data that reveals a protected region containing a distal AcaCD motif (Figure 3D), and supported by previous observations of the promoter-bound RNA polymerase holoenzyme complex footprint between position −54 to +22 relative to the transcription start site [28]. An interesting example of this organization is the regulation of the *vcrx059* and *traI* divergent promoter region, in which two partially overlapping transcription initiation complexes are detected (Figure 3E).

In addition to measuring transcriptome expression in pVCR94Δ*acaCD* and pVCR94Δ*acaCD* complemented with IPTG-induced p*acaDC* ^3×FLAG^, we also performed RNA-seq on wild-type pVCR94 (Dataset S1). Our results indicate little differences between the transcriptome expression levels of wild-type pVCR94 and p*acaDC* ^3×FLAG^-complemented pVCR94Δ*acaCD* (Pearson coefficient of 0.87). On the contrary, these two transcriptomes are clearly different from pVCR94Δ*acaCD* (Pearson coefficients of 0.12 and −0.04, respectively). In total, pVCR94 and p*acaDC* ^3×FLAG^-complemented pVCR94Δ*acaCD* share 76 differentially expressed genes out of 88 when compared to pVCR94Δ*acaCD*, which further supports the high similarity between their gene expression profiles (Dataset S1). These findings suggest that AcaCD in wild-type pVCR94 is at least partially active in *E. coli* under laboratory conditions because an appropriate activating signal is already being sensed and/or the expression of *acaC* and *acaD* is not efficiently repressed. This hypothesis is consistent with the fact that *acr1* and *acr2* negatively regulate conjugative transfer efficiency while not completely abolishing it (Figure 1B). Our results strikingly contrast with the conclusion drawn by others that most of the backbone of IncA/C plasmid pAR060302, including *tra* genes, is transcriptionally inactive in *E. coli* [35]. This interpretation is puzzling given the very high nucleotide identity between core regions of IncA/C plasmids, that reads per kilobase per million reads (RPKM) expression values reported for pAR060302 genes expressed at a “low level” such as *repA* reach several thousand units [35], and that pAR060302 is self-transferable at high frequencies by conjugation under laboratory conditions similar to ours [22].

### AcaCD drives the mobility of two unrelated classes of genomic islands

**Figure 4:**
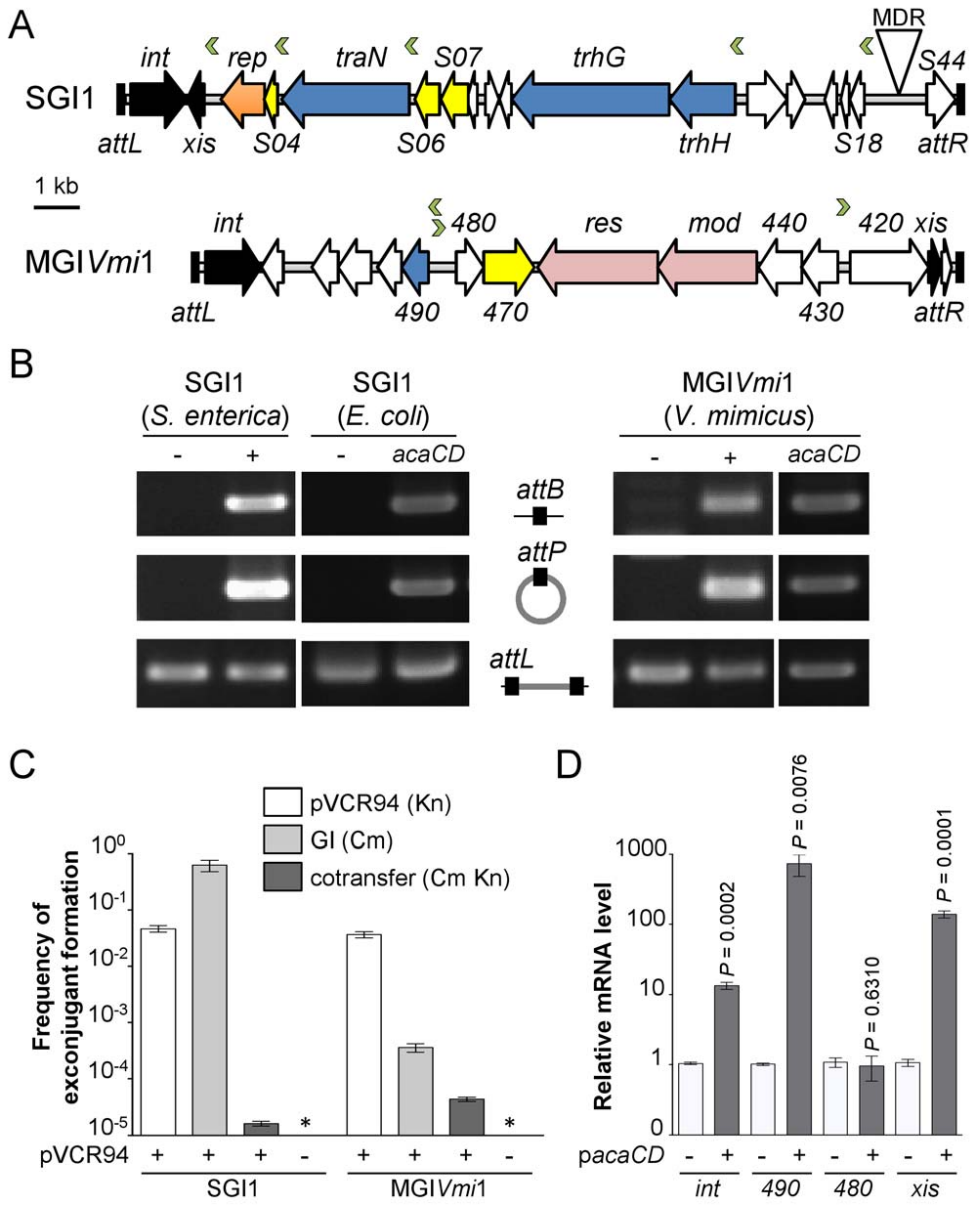
IncA/C-dependent excision and transfer of GIs. (A) Schematic representation of SGI1 from *S. enterica* Typhimurium DT104 and MGI*Vmi*1 from *V. mimicus* VM573. The left and right junctions (*attL* and *attR*) within the host chromosome are indicated. SGI1 (42 596 bp, Genbank AF261825) and MGI*Vmi*1 (16 511 bp, Genbank NZ_ACYV01000005) are integrated into the 3′ end of *trmE* and *yicC* (*attL* sides) in their respective hosts. ORFs with similar function are indicated by colors as follows: black, DNA recombination; orange, DNA replication; blue, conjugative transfer; yellow, regulatory function; pink, putative type III restriction-modification system; white, unknown functions; MDR, multidrug resistance locus. Green chevrons indicate the position and orientation of predicted AcaCD-binding sites (see Table 1). For clarity, ORF names *S0XX* were shortened as *SXX* for SGI1 and VMD_06XXX as XXX for MGI*Vmi*1. (B) AcaCD induces SG1 and MGI*Vmi*1 excision. Excision was detected by PCR on genomic DNA to specifically amplify the *attB* chromosomal site and the *attP* site resulting from the excision of the GIs in *S. enterica* Typhimurium or *E. coli* bearing SGI1 and *V. mimicus* bearing MGI*Vmi*1. Integrated GIs were detected by amplification of the *attL* site. Assays were done in strains devoid of plasmid (−), bearing pVCR94ΔX3 (+) or only expressing *acaCD* (*acaCD*) from p*acaDC* ^3×FLAG^ for assays in *E. coli* or p*acaCD* in *V. mimicus*. (C) Intraspecific mobilization of both GIs was assayed using *E. coli* MG1655 Rf bearing pVCR94ΔX3 and SGI1 or MGI*Vmi*1 as a donor and the otherwise isogenic strain MG1655 Nx as a recipient. Exconjugants were selected for the acquisition of either GI, pVCR94ΔX3, and for cotransfer of both. Transfer frequencies are expressed as the number of exconjugant per donor CFUs. The bars represent the mean and standard deviation values obtained from three independent experiments. The asterisk indicates that the frequency of exconjugant formation was below the detection limit (<10^−8^). (D) AcaCD induces the expression of the putative excision and mobilization genes of MGI*Vmi*1. Relative mRNA levels of *int* (VMD_06510), *490* (VMD_06490), *480* (VMD_06480) and *xis* (VMD_06410) were measured by RT-qPCR assays on cDNA of *V. mimicus* VM573 devoid of plasmid (−) or expressing *acaCD* from p*acaCD* (+). The bars represent the mean and standard deviation values obtained from three independent experiments. Comparison between the strain expressing or not AcaCD were done using two-tailed Student's *t*-tests and the *P*-values are indicated above to the bars.

Determination of the AcaCD-bound DNA motif provides the opportunity to investigate the impact of IncA/C plasmids on genome dynamics. For instance, IncA/C plasmids are known to mobilize SGI1 in *S. enterica* by a yet uncharacterized mechanism [14], [15], [36]. SGI1 elements confer and propagate MDR in various pathogenic bacteria [16], [17], [37]. Data mining using the AcaCD-binding motif revealed putative sites upstream of the genes *xis*, *S004*/*rep*, *traN* and *trhH/trhG* likely involved in the mobility (recombination directionality factor and mobilization genes) of SGI1 (Figure 4A and Table 1). We carried out a similar analysis on the sequenced genome of *V. mimicus* VM573 and identified three AcaCD-binding motifs upstream of three genes, two of which coding for a predicted recombination directionality factor (VMD_06410, *xis*) and a distant homolog of Vcrx001 (VMD_06490, 29% of identity over 85 amino acid residues), which is a conserved key factor for conjugative transfer of IncA/C plasmids (Table 1) [19]. The third gene, VMD_06480, has no predicted function. The three genes are part of an unannotated 16 511-bp GI integrated into the 3′ end of *yicC*, that we named MGI*Vmi*1 (Figure 4A and Table 1). MGI*Vmi*1 could confer adaptive traits to its host, notably resistance to bacteriophages conferred by a putative type III restriction-modification (*res*-*mod*) (Figure 4A). SGI1 and MGI*Vmi*1 are prototypes of two families of GIs that are phylogenetically unrelated to each other and to IncA/C plasmids.

**Table 1:** Prediction of putative AcaCD-binding sites in SGI1 and MGI*Vmi*1.

Element	Strand	Start	End	*p*-value^a^	Matched Sequence^b^	Downstream gene(s)
SGI1	−	2016	2043	3.2e-10	**TTCGCGCCCTAAAAGGGCAGATCCAGAG**	*xis*
SGI1	−	3541	3568	1.2e-11	**TAGGTGCCCAAATAGGGCACTTCCAGAG**	*S004*/*rep*
SGI1	−	6444	6471	6.1e-06	**CGTATGCCGCAAAAGGGCAAATAGCGAT**	*traN*
SGI1	−	13484	13511	7.9e-09	**GGACTGCCCAAATTGGACAGTTTGGGAG**	*trhH*/*trhG*
SGI1	−	16360	16387	2.6e-12	**AATGTGCCCAAAAAGGGCCAATACAGCG**	*S018*
MGI*Vmi*1	+	6832	6859	1.7e-07	**AAAATGCCCAATCAGGACAGTTTGAGCG**	VMD_06480/06470
MGI*Vmi*1	−	6827	6854	7.8e-06	**AAACTGTCCTGATTGGGCATTTTCGCAG**	VMD_06490
MGI*Vmi*1	+	15770	15797	2.4e-06	**TTCAGTACCAAAATGGGCAATTACAGAT**	VMD_06420/*xis*

^*a*^Only output matches with *p*-values below 1.0e-05 are shown and considered as significant hits.

^*b*^Matches were predicted by FIMO (MEME suite) based on the AcaCD logo presented in Figure 3C.

Based on these observations, we hypothesized that expression of *acaCD* either from pVCR94 or from pBAD30 would trigger the excision of both SGI1 and MGI*Vmi*1 from their respective host chromosome. To verify this hypothesis, we monitored by PCR amplification the formation of a chromosomal *attB* site and of an *attP* site resulting from the circularization of the two GIs (Figure 4B). While no spontaneous excision of either GI was detected in the absence of pVCR94, both GIs excised from the chromosome not only in the presence of pVCR94 but also upon ectopic expression of *acaCD* in cells lacking pVCR94 (Figure 4B). Finally, to confirm that IncA/C plasmids can mobilize the novel GI MGI*Vmi*1, we carried out inter- and intraspecific mating assays using pVCR94 as the helper plasmid and a modified MGI*Vmi*1 carrying *res*::*cat* (Cm resistant) as a selectable marker. As a positive control, we used SGI1 and took advantage of its natural selectable markers. For both SGI1 and MGI*Vmi*1, no exconjugant were observed in the absence of pVCR94 (Figure 4C). In contrast, both elements were specifically mobilized in its presence. On one hand, transfer of SGI1 occurred at very high frequency (almost all recipient cells received a copy of SGI1), but co-acquisition of pVCR94 was infrequent. This observation suggests that negative interactions may exist between these mobile genetic elements. On the other hand, MGI*Vmi*1 transferred at a lower rate than pVCR94 (Figure 4C). We confirmed AcaCD-specific expression of the MGI*Vmi*1-borne genes *xis* and VMD_06490 by real-time-quantitative PCR (RT-qPCR) (Figure 4D). *int* mRNA level was also specifically increased by AcaCD, despite the lack of a predicted AcaCD-binding motif in its promoter region. This observation suggests that its enhanced expression could be driven by the promoter upstream of *xis* on a large transcript containing *attP* on the circular excised form of MGI*Vmi*1. For a reason that remains to be established and despite the presence of a predicted AcaCD-binding motif, VMD_06480 expression was not regulated by AcaCD.

Unlike SGI1, which seem to encode a large set of proteins likely dedicated to mobilization (TraN, TrhG and TrhH), MGI*Vmi*1 only encodes a distant homolog of Vcrx001 (VMD_06490), which is strongly induced by AcaCD (Figure 4D). Based on these differences, we speculate that MGI*Vmi*1 is not as efficient as SGI1 to hijack the conjugative apparatus encoded by IncA/C plasmids. Characterization of the genetic and molecular mechanisms of SGI1 and MGI*Vmi*1 mobilization by IncA/C helper plasmids are ongoing.

**Figure 5:**
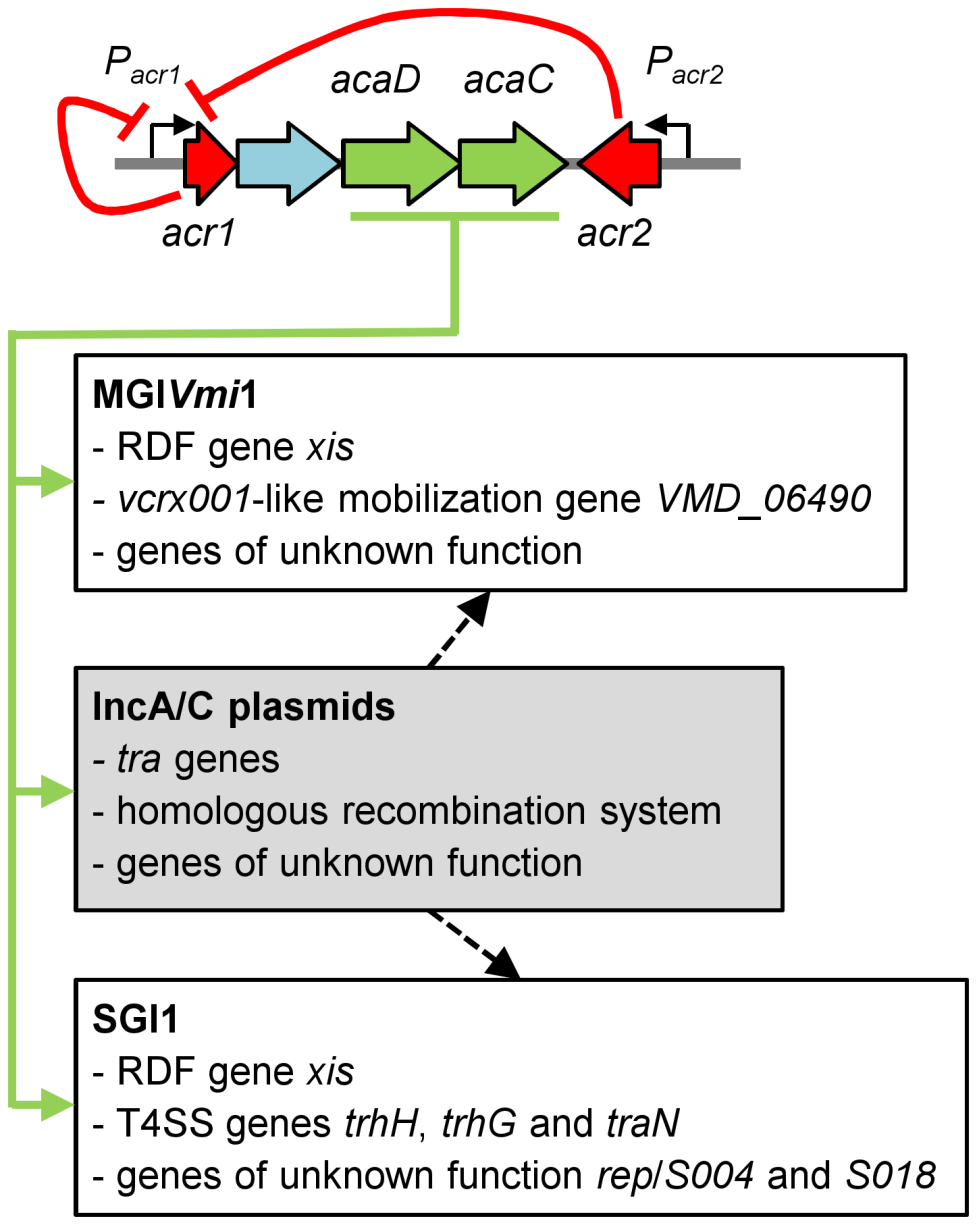
Model of regulation of IncA/C plasmids and interaction with genomic islands. Expression of the master activator complex AcaCD from the promoter of *acr1* (*P_acr1_*) is directly repressed by Acr1 and Acr2 (red arrows). AcaCD directly activates the expression of the transfer genes of pVCR94, as well as the expression of the *bet/exo* homologous recombination system and numerous genes of unknown function (green arrows). AcaCD triggered the excision of SGI1 and MGI*Vmi*1 by directly activating the expression of the RDF genes *xis*. AcaCD activates the expression of the *vcrx001*-like mobilization gene and other genes of unknown function in MGI*Vmi*1. AcaCD also activates the expression of three genes coding for putative component of type IV secretion system (T4SS), as well as genes of unknown function such as *S004*, *rep* and *S018* in SGI1. IncA/C plasmids likely provide additional functions for genomic islands dissemination such as *oriT* recognition and processing or formation of the mating pore (black hatched arrows). Genes are color-coded as in Figure 1A.

Altogether, these results unraveled a novel example of the multiple and intricate interactions linking phylogenetically unrelated mobile genetic elements. Moreover, our study provides new evidence that many GIs are not defective for their propagation but rather mobilizable GIs, whose biology is just linked and perfectly adapted to other self-transmissible helper elements [15], [38], [39], [40] (Figure 5).

### Concluding remarks

IncA/C conjugative plasmids and SXT/R391 ICEs both rely on transcriptional activator complexes that are reminiscent of the FlhDC master activator of flagellar genes to enable expression of their *tra* genes. Nevertheless, AcaCD recognizes DNA motifs that are unrelated to the recognition motif of FlhDC [25]. Moreover, AcaCD and SetCD, the master activator of SXT/R391 ICEs, are not exchangeable and are thus expected to recognize completely unrelated DNA motifs. Our study revealed that the AcaCD regulon extends beyond the sole genes involved in the dissemination of IncA/C plasmids and that AcaCD acts as a beacon signaling the presence of a helper plasmid, allowing the propagation of two unrelated classes of GIs. Similarly, SetCD has been shown to trigger the excision of MGIs originating from various pathogenic *Vibrio* species [38], [39], [41], thereby suggesting that sequences bound by AcaCD- and SetCD-like activators can be easily mimicked by unrelated GIs to regulate their own gene expression. Clearly, our results and observations from others indicate that GIs are not necessarily defective or decaying mobile genetic elements, unable to propagate. Instead, most are likely quiescent parasites awaiting opportunities to hijack helper self-transmissible elements using diverse strategies [14], [38], [42]. Such intricate connections between various genetic elements support their major impact on the evolution of genomes and on the adaptation of bacteria to their environment, particularly in the current context of massive emergence of multidrug resistant pathogens worldwide. Ultimately, future research investigating the regulation of other mobile genetic elements relying on similar transcriptional activator complexes to regulate their own dissemination will unravel unforeseen regulatory networks linking self-transmissible and mobilizable elements.

## Materials and Methods

### Bacterial strains and bacterial conjugation assays

The bacterial strains used in this study are described in Table 2. The strains were routinely grown in Luria-Bertani (LB) broth at 37°C in an orbital shaker/incubator and were maintained at −80°C in LB broth containing 15% (vol/vol) glycerol. Antibiotics were used at the following concentrations: ampicillin (Ap), 100 µg/ml; chloramphenicol (Cm), 20 µg/ml for *E. coli*, 30 µg/ml for *S. enterica* and 10 µg/ml for *V. mimicus*; erythromycin (Em), 10 µg/ml; kanamycin (Kn), 50 µg/ml or 10 µg/ml for single copy integrants of pOPlacZ; nalidixic acid (Nx), 40 µg/ml; rifampicin (Rf) 50 µg/ml; spectinomycin (Sp), 50 µg/ml; streptomycin (Sm), 200 µg/ml; sulfamethoxazole (Su), 160 µg/ml; tetracycline (Tc), 12 µg/ml; trimethoprim (Tm), 32 µg/ml. Conjugation assays were performed as described elsewhere [19]. To induce expression from pBAD30 and from pAH56 in complementation assays, mating experiments were carried on LB agar plates supplemented with 0.02% L-arabinose or 0.1 mM isopropyl β-D-1-thiogalactopyranoside (IPTG), respectively.

**Table 2:** Strains and plasmids used in this study.

Strains or plasmids	Relevant genotype or phenotype	References
*Escherichia coli*		
BW25113	F^−^, Δ(*araD-araB*)*567*, Δ*lacZ4787*(*::rrnB-3*), λ–, *rph-1*, Δ(*rhaD-rhaB*)*568*, *hsdR514*	[50]
BW25113 Nx	Nx^r^-derivative of BW25113	[19]
MG1655 Nx	Nx^r^-derivative of MG1655	[51]
MG1655 Rf	Rf^r^-derivative of MG1655	[51]
BL21(DE3)	F^−^, *ompT* Δ*hsdS gal dcm* (DE3)	Novagen
β2163	(F^−^) RP4-2-Tc::Mu *ΔdapA*::(*erm*-*pir*) (Kn Em)	[49]
*Salmonella enterica*		
St038	serovar Typhimurium; SGI1^+^ (Ap Cm Tc Su Sp Sm)	F. Malouin
*Vibrio mimicus*		
VM573	Patient with diarrhea; USA, 1990s; CT^+^ TCP^+^ MGI*Vmi*1^+^ (Ap Sp Su)	[68]
Plasmids		
pVCR94	IncA/C conjugative plasmid; *V. cholerae* O1 F1939; Rwanda, 1994	[19]
pVCR94ΔX	Su^r^-derivative of pVCR94 (Su)	[19]
pVCR94ΔX2	Sp^r^-derivative of pVCR94ΔX (Sp Su)	This study
pVCR94ΔX3	Kn^r^-derivative of pVCR94ΔX (Kn Su)	This study
pVCR94Δ*25*	pVCR94 ΔX2 Δ*vcrx025* (Sp Su)	This study
pVCR94Δ*acaC*	pVCR94 ΔX2 Δ*acaC* (Sp Su)	This study
pVCR94Δ*acaD*	pVCR94 ΔX2 Δ*acaD* (Sp Su)	This study
pVCR94Δ*acaCD*	pVCR94 ΔX2 Δ*acaCD* (Sp Su)	This study
pVCR94Δ*acr1*	pVCR94 ΔX2 Δ*acr1* (Sp Su)	This study
pVCR94Δ*acr2*	pVCR94 ΔX2 Δ*acr2* (Sp Su)	This study
pBAD30	*ori*_p15A_ *araC P*_BAD_ (Ap)	[45]
p*acaC*	pBAD30::*acaC* (Ap)	This study
p*acaD*	pBAD30::*acaD* (Ap)	This study
p*acaCD*	pBAD30::*acaCD* (Ap)	This study
p*acr1*	pBAD30::*acr1* (Ap)	This study
p*acr2*	pBAD30::*acr2* (Ap)	This study
pGG2B	pBAD30::*setDC* (Ap)	[69]
pSW23T	*oriT*_RP4_; *oriV*_R6Kγ_ (Cm)	[49]
pAH56	*oriV*_R6Kγ_; *attP*_λ_; *lacI*; *P*_tac_ (Kn)	[70]
pJL148	SPA-tag (CBP-TEV site-3×FLAG) (Kn)	[47]
p*acaDC* ^3×FLAG^	pAH56::*acaDC* ^3×FLAG^ (Kn)	This study
p*setDC* ^3×FLAG^	pAH56::*setDC* ^3×FLAG^ (Kn)	This study
pOP*lacZ*	pAH56 *lacZ* (Kn)	This study
pProm*traL*	pOP*lacZ P_traL_*-*lacZ* (Kn)	This study
pProm*traI*	pOP*lacZ P_traI_*-*lacZ* (Kn)	This study
pProm*traF*	pOP*lacZ P_traF_*-*lacZ* (Kn)	This study
pProm*acr1*	pOP*lacZ P_acr1_*-*lacZ* (Kn)	This study
pProm*152*	pOP*lacZ P_152_*-*lacZ* (Kn)	This study
pINT-ts	*oriR101*; *cI857*; λ*p*_R_-*int*_λ_ (Ap Ts)	[70]
pET-24b(+)	*oriV*_pBR322_; *lacI*; *P*_T7_; 6×His; expression vector (Kn)	Novagen
p*acaDC* ^6×His^	pET-24b(+)::*acaDC* ^6×His^ (Kn)	This study
pSIM6	λRed recombination thermo-inducible encoding plasmid (Ts Ap)	[52]
pKD3	PCR template for one-step chromosomal gene inactivation (Cm)	[50]
pKD13	PCR template for one-step chromosomal gene inactivation (Kn)	[50]
pVI36	PCR template for one-step chromosomal gene inactivation (Sp)	[51]
pCP20	Flp recombinase thermo-inducible encoding plasmid (Ts Ap Cm)	[53]

Ap, ampicillin; Cm, chloramphenicol; Em, erythromycin; Kn, kanamycin; Nx, nalidixic acid; Sp, spectinomycine; Rf, rifampicin; Sm, streptomycine; Su, sulfamethoxazole; Tc, tetracycline; Tm, trimethoprim; Ts, thermosensitive; CT, cholera toxin; TCP, toxin co-regulated pilus.

### Molecular biology methods

Genomic and plasmid DNA were prepared using the Wizard Genomic DNA Purification Kit (Promega) and EZ-10 Spin Column Plasmid DNA Minipreps Kit (Biobasic), respectively, according to manufacturer's instructions. All the enzymes used in this study were purchased from New England BioLabs or Enzymatics. PCR assays were performed with the primers described in Table S4. The PCR conditions were as follows: (i) 3 min at 94°C; (ii) 30 cycles of 30 sec at 94°C, 30 sec at the appropriate annealing temperature, and 1 minute/kb at 68°C; and (iii) 5 min at 68°C. When necessary, PCR products were purified using a EZ-10 Spin Column PCR Products Purification Kit (Biobasic) according to manufacturer's instructions. *E. coli* was transformed by electroporation according to Dower *et al.* [43]. *V. mimicus* was transformed by electroporation according to Occhino *et al.* [44] with modified G buffer (200 mM sucrose, 1 mM Hepes, pH 7.5). Electroporation was carried out in a BioRad GenePulser Xcell apparatus set at 25 µF, 200 V and 1.8 kV using 1-mm gap electroporation cuvettes. Sequencing reactions were performed by the Plateforme de Séquençage et de Génotypage du Centre de Recherche du CHUL (Québec, QC, Canada).

### Plasmids and strains construction

Plasmids and oligonucleotides used in this study are listed in Table 2 and S4. Complementation vectors derived from the pBAD30 vector [45]. The ORFs *acr1*, *acr2*, *acaC*, *acaD* and *acaCD* were amplified using primers pairs vcrx146.for/vcrx146.rev, vcrx150.for/vcrx150.rev, vcrx149(acaC).for/vcrx149(acaC).rev, vcrx148(acaD).for/vcrx148(acaD).rev, vcrx148(acaD).for/vcrx149(acaC).rev and cloned into the EcoRI restriction site of pBAD30 to generate p*acr1*, p*acr2*, p*acaC*, p*acaD* and p*acaCD*, respectively. The transcriptional fusion vector pOPlacZ contains the promoterless *lacZ* gene from *E. coli* K12 MG1655 with its native Shine-Dalgarno sequence. This plasmid was constructed by amplifying the *lacZ* gene using primer pair Op-lacZ-F/Op-lacZ-R and subsequent cloning into the 2 462-bp fragment of PstI-digested pAH56 using the Gibson assembly method [46]. PCR fragments containing the promoter region upstream of *acr1*, *traL*, *traI*, *traF* and *vcrx152* were cloned into the PstI restriction site of pOPlacZ to produce pProm*acr1*, pProm*traL*, pProm*traI*, pProm*traF* and pProm*152*, respectively. The vectors p*acaDC* ^3×FLAG^ and p*setDC* ^3×FLAG^ used for complementation assays and ChIP-exo experiments derived from pAH56. Briefly, the *acaDC* and *setDC* loci were amplified using primer pairs acaDF-NdeI/acaCR-HindIII and setDF-NdeI/setCR-HindIII, respectively, and cloned into pCR2.1 (Invitrogen) to generate pCR2.1::*acaDC* and pCR2.1::*setDC*. 3×FLAG was amplified from pJL148 [47] using the primer pair FlagF/FlagR and subsequently cloned into the HindIII site of pCR2.1::*acaDC* and pCR2.1::*setDC* to generate pCR2.1::*acaDC* ^3×FLAG^ and pCR2.1::*setDC* ^3×FLAG^. The *acaDC* ^3×FLAG^ and *setDC* ^3×FLAG^ inserts were recovered by NdeI/SalI digestion and subsequently cloned into the NdeI/SalI-digested pAH56 to generate p*acaDC* ^3×FLAG^ and p*setDC* ^3×FLAG^ [48]. p*acaDC* ^6×His^ was obtained by cloning of *acaDC* amplified with acaDF-NdeI/acaCR-HindIII into pET-24b(+) (Novagen). pRes for insertion of a Cm marker into MGI*Vmi*1 was obtained cloning into EcoRI/BamHI-digested pSW23T [49] the 732-bp PCR fragment amplified using the primer pair GIVmi-res2F/GIVmi-res2R on genomic DNA of *V. mimicus* VM573.

Deletion mutants of pVCR94ΔX were constructed using the one-step chromosomal gene inactivation technique and are listed in the Table 1 [19], [50]. Primers used are listed in Table S4. The pVCR94ΔX derivatives pVCR94ΔX2 (Sp) and pVCR94ΔX3 (Kn) were constructed using primer pair 94DelXnoFRT.for/94DelXnoFRT.rev and pVI36 and pKD13 as templates, respectively [50], [51]. Subsequent deletions of *vcrx025*, *acr1*, *acr2*, *acaC*, *acaD* and *acaCD* were done on pVCR94ΔX2 using primer pairs 94Delvcrx025.for/94Delvcrx025.rev, 94Delvcrx146.for/94Delvcrx146.rev, 94Delvcrx150.for/94Delvcrx150.rev, 94DelacaC.for/94DelacaC.rev, 94DelacaD.for/94DelacaD.rev and 94DelacaD.for/94DelacaC.rev, respectively, and pKD3 as the template. The λRed recombination system was expressed using pSIM6 as described by Datta *et al.* [52]. When possible, the antibiotic resistance cassette was removed from the resulting construction by Flp-catalyzed excision using the pCP20 vector [53]. All deletions were designed to be non-polar and verified by PCR and antibiotic resistance profiling.

 MGI*Vmi*1 was labelled with a Cm resistance marker in *V. mimicus* VM573 by inserting the pSW23T-derivative suicide plasmid pRes into the putative type III restriction gene *res*. Briefly, pRes was mobilized from *E. coli* β2163 to *V. mimicus* VM573 and exconjugants were selected on LB agar plates supplemented with 10 µg/ml chloramphenicol in the absence of DL-α,ε-diaminopimelic acid according to Demarre *et al.* [49].

### Proteins purification and analysis

*E coli* BL21(DE3) carrying p*acaDC^6×His^* was grown to OD_600 nm_ = 0.5 and induced for 2 hours with 1 mM of IPTG. Purification of AcaC tagged with a 6×His C-terminal epitope was done using a Ni-NTA affinity chromatography following the manufacturer's instructions (Qiagen). Migration of the purified proteins on 12% SDS-PAGE gel and Western blotting experiments using anti-6×His tag antibody were done as described by the manufacturer (Life Technologies/Invitrogen). Protein identification was performed at the proteomics platform of the Université de Sherbrooke on an excised acrylamide gel slice digested with trypsin and subjected to LC/MS/MS analysis as previously described [54].

### ChIP-exo experiments and RNA sequencing

A thorough description of the chromatin immunoprecipitation coupled with exonuclease digestion and RNA sequencing procedures is given in Text S1 and Table S5.

### RNA isolation and qRT-PCR

Cells from *V. mimicus* VM573 that contain no vector or p*acaCD* were recovered after 2.5 hours of induction with 0.02% of arabinose. Total RNA extraction was done using an RNeasy minikit (Qiagen) following the manufacturer's instructions. Purified RNA samples were subsequently subjected to gDNA digestion using Turbo DNase (Ambion) following the manufacturer's instructions. RNA integrity was assessed using an Agilent 2100 Bioanalyzer (Agilent Technologies). qRT-PCR assays were performed on the RNomics platform of the Laboratoire de Génomique Fonctionnelle de l'Université de Sherbrooke (http://lgfus.ca). Reverse transcription was performed on 2.2 µg total RNA with Transcriptor reverse transcriptase, random hexamers, dNTPs (Roche Diagnostics), and 10 units of RNAseOUT (Invitrogen Life Technologies) following the manufacturer's protocol in a total volume of 20 µl. All forward and reverse primers were individually resuspended to 20–100 µM stock solutions in Tris-EDTA buffer (IDT) and diluted as a primer pair to 1 µM in RNase DNase-free water (IDT). Quantitative PCR (qPCR) reactions were performed in 10-µl volumes in 96 well plates on a CFX-96 thermocycler (Bio-Rad) with 5 µL of 2× iTaq Universal SYBR Green Supermix (Bio-Rad), 10 ng (3 µl) cDNA, and 200 nM final (2 µl) primer pair solutions. The following cycling conditions were used: 3 min at 95°C; 50 cycles: 15 s at 95°C, 30 s at 60°C, 30 s at 72°C. Relative expression levels of *int* (VMD_06510), VMD_06490, VMD_06480 and *xis* (VMD_06410) were calculated using a model taking into account multiple stably expressed reference genes [55] and housekeeping genes *rpoZ* and *gyrA* evaluated by geNorm [56]. Primer design (see Table S4) and validation were evaluated as described elsewhere [57]. In every qPCR run, a no-template control was performed for each primer pair and a no-reverse transcriptase control was performed for each cDNA preparation. Experiments were carried out three times on three biological replicates and combined.

### Detection of SGI1 and MGI*Vmi*1 excision

Excision of the GIs was detected by PCR on genomic DNA of the strains containing either SGI1 (*S. enterica* or *E. coli*) or MGI*Vmi*1 (*V. mimicus*) using the primers listed in the Table S4. For SGI1, the *attL* site was amplified using primer pair SGI-1attL.for/SGI-1attL.rev in *S. enterica* and EcU7-L12.for/SGI-1attL.rev in *E. coli* [15]. The chromosomal site *attB* was detected using SGI-1attL.for/SGI-1attR.rev in *S. enterica* and EcU7-L12.for/Ec104D.rev in *E. coli*. The *attP* site carried by the extrachromosomal circular form of the element was amplified using the primer pair SGI-1attL.rev/SGI-1attR.for. Based on the same methodology for MGI*Vmi*1 in *V. mimicus*, primer pairs GIVmi-A/GIVmi-B, GIVmi-A/GIVmi-D and GIVmi-B/GIVmi-C were used to detect *attL*, *attB* and *attP*, respectively.

### Phylogenetic analyses

The molecular phylogenetic analysis of the *acr1*-*vcr147*-*acaDC*-*acr2* locus was conducted in MEGA6 [58]. The nucleotide sequence of the 2452-bp sequence of pVCR94 starting at the initiation codon of *acr1* and ending at the initiation codon of *acr2* was used to search for homologous sequences in the Genbank Nucleotide collection (nr/nt) database using Megablast [59]. Phylogenetic analyses were computed using a nucleotide alignment generated by MUSCLE [60]. The evolutionary history was inferred by using the Maximum Likelihood method. Initial tree(s) for the heuristic search were obtained automatically by applying Neighbor-Join and BioNJ algorithms to a matrix of pairwise distances estimated using the Maximum Composite Likelihood (MCL) approach, and then selecting the topology with superior log likelihood value. Identical procedures were used for the molecular phylogenetic analysis of the 1101-bp *repA* gene of pVCR94 (Figure S1).

### Data availability

Fastq files for each experiment were deposited at the NCBI Sequence Read Archive (SRA) under accession numbers SRX675564 and SRR1544064 for ChIP-exo, SRX675582 and SRR1544143 for total RNAseq as well as SRX675814 and SRR1544479 for 5′-RACE. Complete data from aligned reads for ChIP-exo and RNA-seq can also be visualized using the UCSC genome browser at http://bioinfo.ccs.usherbrooke.ca/pVCR94.html.

## Supporting Information

Figure S1 Molecular phylogenetic analysis of the *repA* replication initiation gene by Maximum Likelihood method. The evolutionary history was inferred by using the Maximum Likelihood method based on the Hasegawa-Kishino-Yano model [67]. The tree with the highest log likelihood (−2386.8588) is shown. The percentage of trees in which the associated taxa clustered together is shown next to the branches. A discrete Gamma distribution was used to model evolutionary rate differences among sites (5 categories (+G, parameter = 0.2014)). The tree is drawn to scale, with branch lengths measured in the number of substitutions per site. The analysis involved 45 nucleotide sequences. Codon positions included were 1st+2nd+3rd+Noncoding. There were a total of 1101 positions in the final dataset. Evolutionary analyses were conducted in MEGA6 [58]. The background color of each leaf indicates the original host species from which each plasmid was isolated. *, XNC1_p lacks the *acr1*-*vcrx147*-*acaDC*-*acr2* regulation cluster.
(TIF)

Figure S2 The 3×FLAG epitope does not alter the function of AcaCD. The donor strain *E. coli* MG1655 Rf was used to transfer pVCR94ΔX or its Δ*acaCD* mutant (Su Sp) to the recipient strain MG1655 Nx. AcaCD and SetCD complementation assays were carried out with the native D subunit and the C subunit C-terminally fused to the 3×FLAG epitope expressed from an IPTG-inducible promoter (*P_tac_*) provided by pAH56, i.e. p*acaDC* ^3×FLAG^ (*acaDC* ^3×FLAG^), p*setDC* ^3×FLAG^ (*setDC* ^3×FLAG^) or empty pAH56 (0) integrated into the *attB*_λ_ locus. Exconjugants were selected as Nx Su Sp colonies. The bars represent the mean and standard deviation values obtained from at least 3 biological replicates. The asterisks indicate that the frequency of transfer was below the limit of detection (<10^−8^).
(TIF)

Figure S3 Comparison of the genetic context of genes coding for AcaCD and SetCD orthologs in IncA/C plasmids and SXT/R391 ICEs. Schematic representation of regulatory regions of pVCR94 from *V. cholerae* O1 El Tor (NC_023291.1) and SXT from *V. cholerae* O139 (AY055428.1). Arrows of similar color represent genes predicted to have similar functions: green, transcriptional activator; yellow, putative transcriptional regulator; red, transcriptional repressor; blue, conjugative transfer; light blue, putative lytic transglycosylase; orange, replication; black, site-specific recombination; white, unknown function. Blue stars indicate the position of origins of transfer (*oriT*). The orange star indicates the position of the origin of replication (*oriV*) of pVCR94 based on identity with pRA1 from *Aeromonas hydrophila* (NC_012885). The black star indicates the position of the *attP* site for chromosomal integration of SXT by site-specific recombination. The percent of identity of orthologous proteins are indicated on dashed lines.
(TIF)

Figure S4 Alignment of AcaCD-dependent promoters in pVCR94. The AcaCD motif is as represented in Figure 3C. AcaCD boxes obtained by MAST analysis are shown in bold green capital letters with their respective *p*-value and downstream regulated gene. The positions of the transcription start sites obtained from 5′-RACE data are indicated in bold blue capital letters and underlined (TSS). Shine-Dalgarno sequences (SD) are underlined while start codons are in capital letters. The approximate positions of the −35 and −10 regions are highlighted in gray. The length of spacers between the represented transcription start sites and the Shine-Dalgarno regions is indicated in base pairs. Since no clear 5′-RACE signal was observed for *vcrx035* and *vcrx098*, the approximate positions of expected transcription start sites are underlined and shown in bold black capital letters.
(TIF)

Table S1 Open reading frames (ORFs) of pVCR94 coding for putative transcriptional regulators.
(DOCX)

Table S2 *acr1*-*vcrx147*-*acaDC*-*acr2* orthologous clusters in IncA/C plasmids.
(DOCX)

Table S3 AcaCD-regulated promoters identified by ChIP-exo and 5′-RACE.
(DOCX)

Table S4 Primers used in this study.
(DOCX)

Table S5 Illumina libraries sequenced in this study.
(DOCX)

Dataset S1 RNAseq transcriptome profiling of wild-type pVCR94, pVCR94Δ*acaCD* and complemented pVCR94Δ*acaCD*.
(XLS)

Text S1 Additional experimental procedures.
(DOCX)
