# Supplementary figures and images for: The Master Activator of IncA/C Conjugative Plasmids Stimulates Genomic Islands and Multidrug Resistance Dissemination
Source: PLoS Genet. 2014 Oct 23;10(10):e1004714. doi: 10.1371/journal.pgen.1004714 (PMC4207636; doi:10.1371/journal.pgen.1004714)

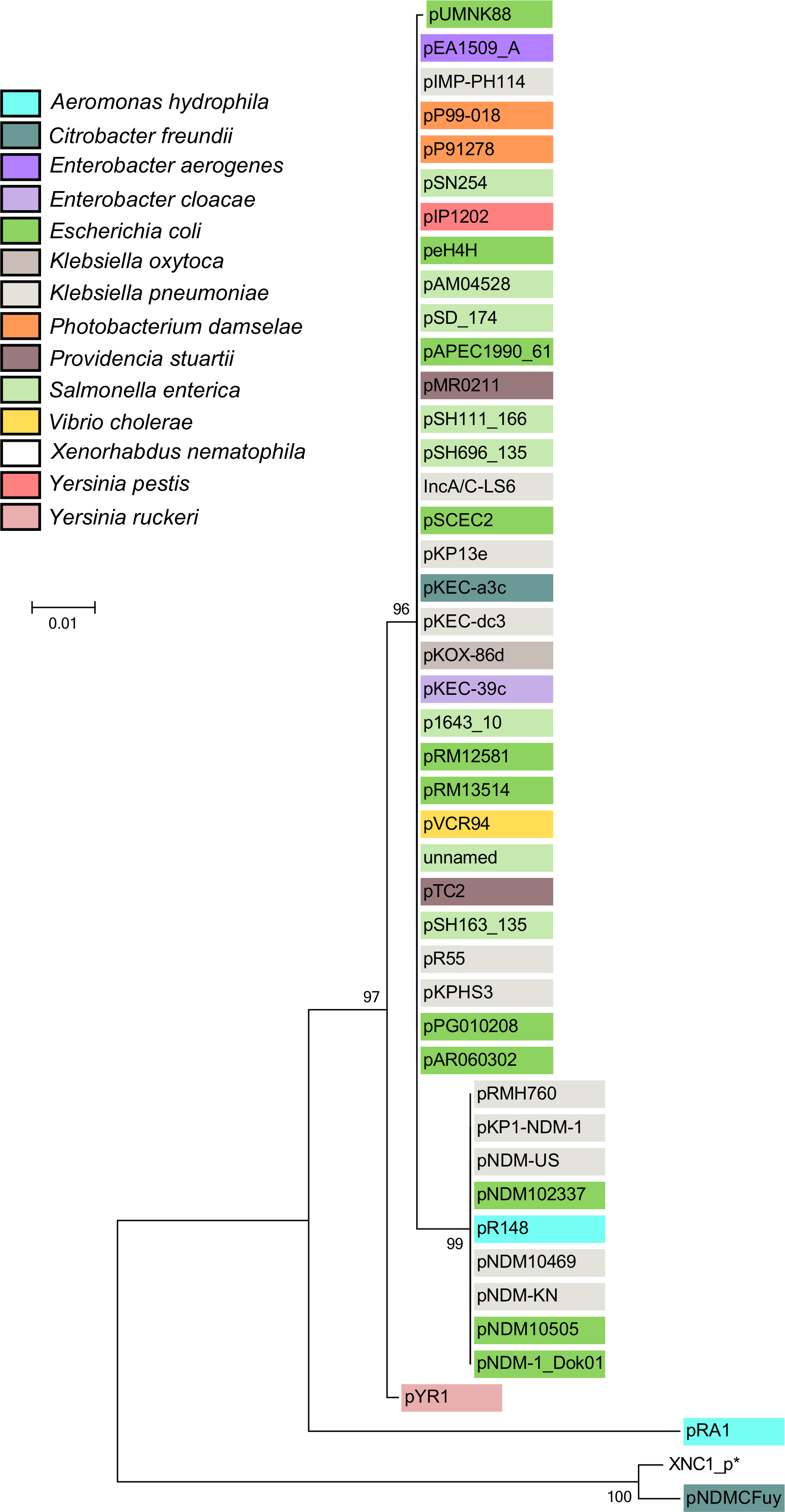

Supplement: Figure S1 — Molecular phylogenetic analysis of the repA replication initiation gene by Maximum Likelihood method. The evolutionary history was inferred by using the Maximum Likelihood method based on the Hasegawa-Kishino-Yano model [67]. The tree with the highest log likelihood (−2386.8588) is shown. The percentage of trees in which the associated taxa clustered together is shown next to the branches. A discrete Gamma distribution was used to model evolutionary rate differences among sites (5 categories (+G, parameter = 0.2014)). The tree is drawn to scale, with branch lengths measured in the number of substitutions per site. The analysis involved 45 nucleotide sequences. Codon positions included were 1st+2nd+3rd+Noncoding. There were a total of 1101 positions in the final dataset. Evolutionary analyses were conducted in MEGA6 [58]. The background color of each leaf indicates the original host species from which each plasmid was isolated. *, XNC1_p lacks the acr1-vcrx147-acaDC-acr2 regulation cluster. (TIF) [file pgen.1004714.s001.tif]

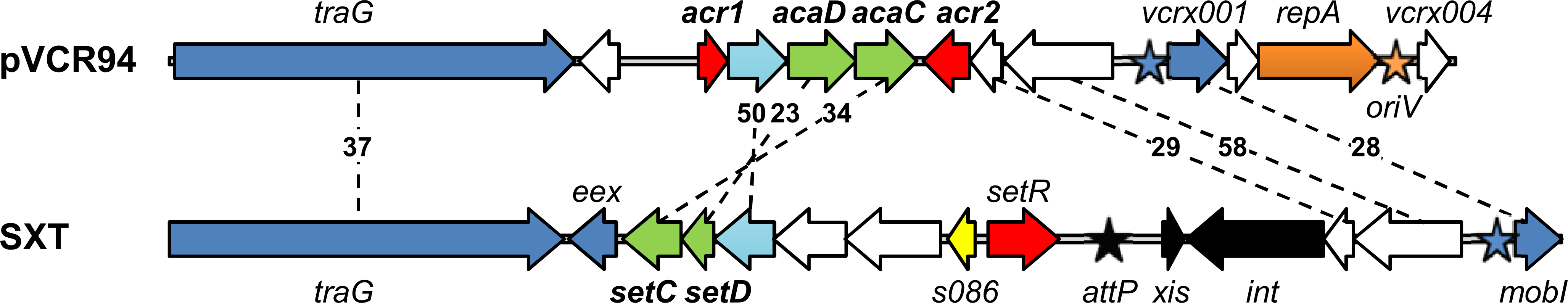

Supplement: Figure S3 — Comparison of the genetic context of genes coding for AcaCD and SetCD orthologs in IncA/C plasmids and SXT/R391 ICEs. Schematic representation of regulatory regions of pVCR94 from V. cholerae O1 El Tor (NC_023291.1) and SXT from V. cholerae O139 (AY055428.1). Arrows of similar color represent genes predicted to have similar functions: green, transcriptional activator; yellow, putative transcriptional regulator; red, transcriptional repressor; blue, conjugative transfer; light blue, putative lytic transglycosylase; orange, replication; black, site-specific recombination; white, unknown function. Blue stars indicate the position of origins of transfer (oriT). The orange star indicates the position of the origin of replication (oriV) of pVCR94 based on identity with pRA1 from Aeromonas hydrophila (NC_012885). The black star indicates the position of the attP site for chromosomal integration of SXT by site-specific recombination. The percent of identity of orthologous proteins are indicated on dashed lines. (TIF) [file pgen.1004714.s003.tif]

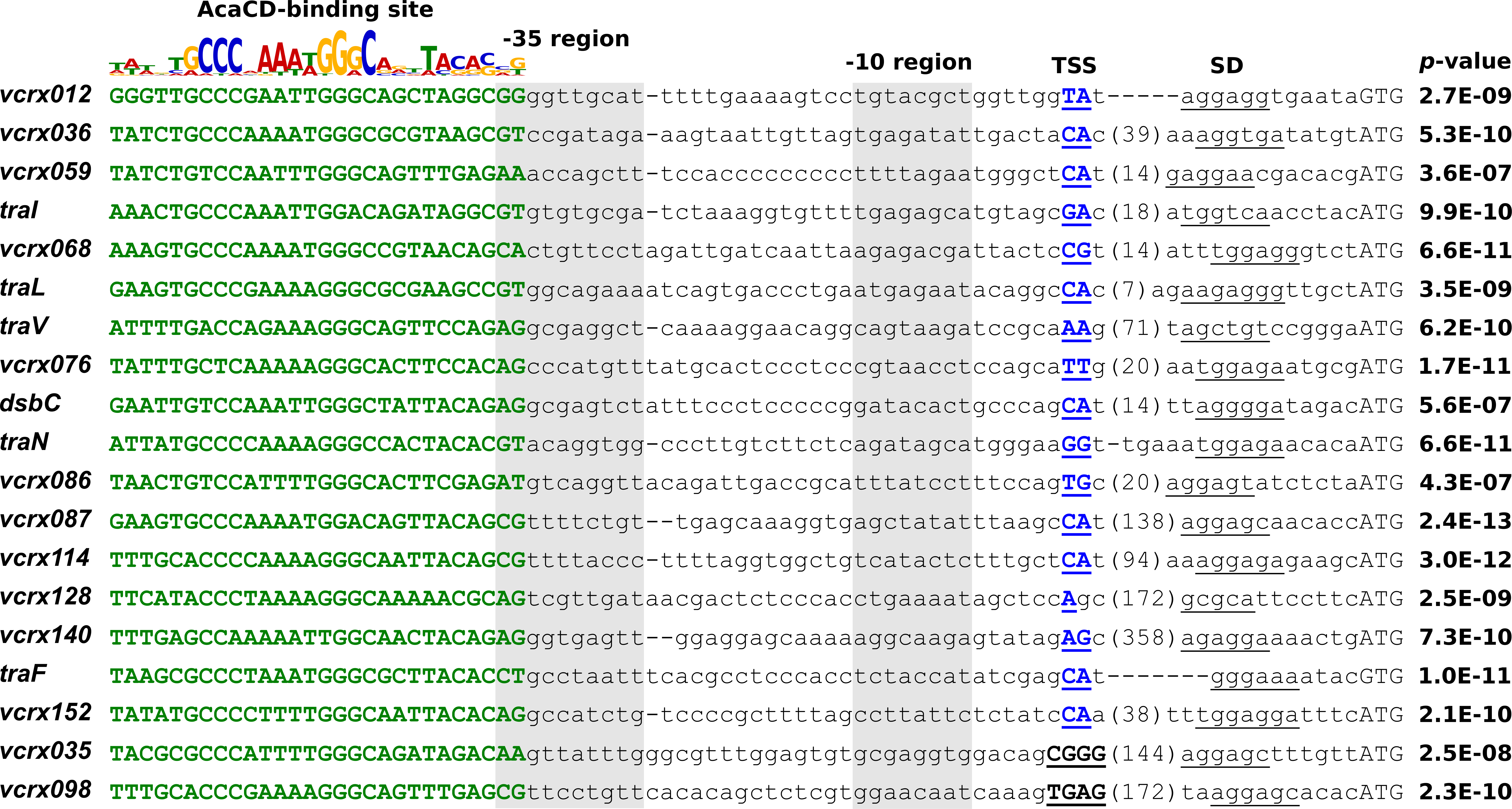

Supplement: Figure S4 — Alignment of AcaCD-dependent promoters in pVCR94. The AcaCD motif is as represented in Figure 3C. AcaCD boxes obtained by MAST analysis are shown in bold green capital letters with their respective p-value and downstream regulated gene. The positions of the transcription start sites obtained from 5′-RACE data are indicated in bold blue capital letters and underlined (TSS). Shine-Dalgarno sequences (SD) are underlined while start codons are in capital letters. The approximate positions of the −35 and −10 regions are highlighted in gray. The length of spacers between the represented transcription start sites and the Shine-Dalgarno regions is indicated in base pairs. Since no clear 5′-RACE signal was observed for vcrx035 and vcrx098, the approximate positions of expected transcription start sites are underlined and shown in bold black capital letters. (TIF) [file pgen.1004714.s004.tif]
