# Supplementary material for: The Master Activator of IncA/C Conjugative Plasmids Stimulates Genomic Islands and Multidrug Resistance Dissemination
Source: PLoS Genet. 2014 Oct 23;10(10):e1004714. doi: 10.1371/journal.pgen.1004714 (PMC4207636; doi:10.1371/journal.pgen.1004714)
Supplement: Table S1 — Open reading frames (ORFs) of pVCR94 coding for putative transcriptional regulators. (DOCX) [file pgen.1004714.s005.docx]

**Table S1.** Open reading frames (ORFs) of pVCR94 coding for putative transcriptional regulators

| **ORF name** | **Size (aa)***^a^* | **Predicted function** | **Most significant Pfam matches** | **Conserved in IncA/C plasmids** |
| --- | --- | --- | --- | --- |
| *vcrx025* | 90 | HUβ-like DNA-binding protein | Bac_DNA_binding (PF00216) | yes |
| *vcrx027* | 100 | Cro-like transcriptional regulator (Xre) | HTH_37 (PF13744) | yes |
| *vcrx049* | 135 | Cd(II)/Pb(II)-responsive transcriptional regulator | MerR_1 (PF13411) | no |
| *vcrx146* (***acr1***) | 90 | Ner-like DNA-binding protein | HTH_35 (PF13693) | yes |
| *vcrx148* (***acaD***) | 202 | FlhD-like transcriptional activator | SUKH-4 (PF14435)*^b^*  FlhD (PF05247)*^b^* | yes |
| *vcrx149* (***acaC***) | 183 | FlhC-like transcriptional activator | FlhC (PF05280) | yes |
| *vcrx150* (***acr2***) | 139 | H-NS-like DNA-binding protein | Histone_HNS (PF00816) | yes |

*^a^* Size in amino-acids of the predicted translation product.

*^b^* These domains were reported as an insignificant Pfam-A match.
