# Supplementary material for: The Master Activator of IncA/C Conjugative Plasmids Stimulates Genomic Islands and Multidrug Resistance Dissemination
Source: PLoS Genet. 2014 Oct 23;10(10):e1004714. doi: 10.1371/journal.pgen.1004714 (PMC4207636; doi:10.1371/journal.pgen.1004714)
Supplement: Table S2 — acr1-vcrx147-acaDC-acr2 orthologous clusters in IncA/C plasmids. (DOCX) [file pgen.1004714.s006.docx]

**Table S2.** *acr1*-*vcrx147*-*acaDC­*-*acr2* orthologous clusters in IncA/C plasmids.

| **Plasmid name** | **gi** | **Accession number** | **Position***^a^* | **Species or strain** |
| --- | --- | --- | --- | --- |
| pR148 | 409729032 | JX141473.1 | 160846-163297 | Aeromonas hydrophila |
| pRA1 | 224831756 | FJ705807.1 | 139862-142313 | Aeromonas hydrophila |
| pKEC-a3c | 619739382 | CP007558.1 | 267237-269688 | Citrobacter freundii CFNIH1 |
| pNDMCFuy | 549084851 | HG428757.1 | 147895-150347 | Citrobacter freundii NDMCF |
| pEA1509_A | 443900801 | FO203354.1 | 157106-159557 | Enterobacter aerogenes EA1509E |
| pKEC-39c | 662716651 | CP008824.1 | 314916-317367 | Enterobacter cloacae ECNIH2 |
| pNDM-1_Dok01 | 345468041 | AP012208.1 | 191638-194089 | Escherichia coli NDM-1 Dok01 |
| pRM12581 | 628088972 | CP007137.1 | 4464-5649 | Escherichia coli O145:H28 RM12581 |
| pRM12581 | 628088972 | CP007137.1 | 1885-3153 | Escherichia coli O145:H28 RM12581 |
| pRM13514 | 573972154 | CP006029.1 | 4463-5648 | Escherichia coli O145:H28 RM13514 |
| pRM13514 | 573972154 | CP006029.1 | 1885-3153 | Escherichia coli O145:H28 RM13514 |
| pAPEC1990_61 | 305670853 | HQ023863.1 | 157159-159610 | Escherichia coli APEC1990_61 |
| pAR060302 | 229561762 | FJ621588.1 | 162608-165059 | Escherichia coli AR060302 |
| peH4H | 229561407 | FJ621586.1 | 144183-146634 | Escherichia coli H4H |
| pNDM10505 | 345105136 | JF503991.1 | 162822-165273 | Escherichia coli N10-0505 |
| pNDM102337 | 430829327 | JF714412.2 | 162052-164503 | Escherichia coli N10-2337 |
| pPG010208 | 305670530 | HQ023861.1 | 131881-134332 | Escherichia coli PG010208 |
| pSCEC2 | 544169431 | KF152885.1 | 130555-133006 | Escherichia coli SCEC2 |
| pUMNK88 | 305670679 | HQ023862.1 | 156651-159102 | Escherichia coli UMNK88 |
| pKOX-86d | 660573358 | CP008790.1 | 188665-191116 | Klebsiella oxytoca KONIH1 |
| IncA/C-LS6 | 514883893 | JX442976.1 | 22901-25352 | Klebsiella pneumoniae |
| pR55 | 377806473 | JQ010984.1 | 166888-169339 | Klebsiella pneumoniae |
| pRMH760 | 596353652 | KF976462.2 | 166691-169142 | Klebsiella pneumoniae |
| pNDM-US | 582035484 | CP006661.1 | 10173-12624 | Klebsiella pneumoniae ATCC BAA-2146 |
| pIMP-PH114 | 555235253 | KF250428.1 | 146789-149240 | Klebsiella pneumoniae CRE114 |
| pKP1-NDM-1 | 591389235 | KF992018.1 | 133616-136067 | Klebsiella pneumoniae KP1 |
| pNDM-KN | 358409925 | JN157804.1 | 62017-64468 | Klebsiella pneumoniae Kp7 |
| pNDM10469 | 378705671 | JN861072.1 | 133891-136342 | Klebsiella pneumoniae N10-0469 |
| pKPHS3 | 365803989 | CP003225.1 | 99091-101542 | Klebsiella pneumoniae subsp. pneumoniae HS11286 |
| pKP13e | 569550126 | CP003998.1 | 67067-69518 | Klebsiella pneumoniae subsp. pneumoniae Kp13 |
| pKEC-dc3 | 640855459 | CP007732.1 | 263274-265725 | Klebsiella pneumoniae subsp. pneumoniae KPNIH27 |
| pP99-018 DNA | 118596719 | AB277723.1 | 113494-115945 | Photobacterium damselae subsp. piscicida |
| pP91278 DNA | 118596907 | AB277724.1 | 107929-110380 | Photobacterium damselae subsp. piscicida |
| pTC2 | 407810043 | JQ824049.1 | 137302-139753 | Providencia stuartii |
| pMR0211 | 374413489 | JN687470.1 | 174355-176806 | Providencia stuartii |
| pAM04528 | 229561581 | FJ621587.1 | 154292-156742 | Salmonella enterica AM04528 |
| pSD_174 | 327536433 | JF267651.1 | 170618-172253 | Salmonella enterica subsp. enterica serovar Dublin 853 |
| pSD_174 | 327536433 | JF267651.1 | 270-790 | Salmonella enterica subsp. enterica serovar Dublin 853 |
| pSH696_135 | 381288584 | JN983048.1 | 131501-133952 | Salmonella enterica subsp. enterica serovar Heidelberg |
| pSH163_135 | 381288102 | JN983045.1 | 131246-133697 | Salmonella enterica subsp. enterica serovar Heidelberg |
| pSH111_166 | 381287804 | JN983043.1 | 21384-23835 | Salmonella enterica subsp. enterica serovar Heidelberg |
| p1643_10 | 657224404 | KF056330.2 | 163689-166140 | Salmonella enterica subsp. enterica serovar Kentucky 1643/10 |
| pSN254 | 133905002 | CP000604.1 | 172552-175002 | Salmonella enterica subsp. enterica serovar Newport SL254 |
| pYT3*^b^* | 544345850 | AB591424.1 | 90796-92305 | Salmonella enterica subsp. enterica serovar Typhimurium |
| unnamed | 523811284 | CP006050.1 | 214790-216540 | Salmonella enterica subsp. enterica serovar Typhimurium var. 5- str. CFSAN001921 |
| unnamed | 523811284 | CP006050.1 | 219066-219775 | Salmonella enterica subsp. enterica serovar Typhimurium var. 5- str. CFSAN001921 |
| pVCR94 | 566131152 | KF551948.1 | 116328-118779 | Vibrio cholerae BI144 |
| pIP1202 | 133905277 | CP000603.1 | 178991-181442 | Yersinia pestis biovar Orientalis IP275 |
| pYR1 | 133904815 | CP000602.1 | 154115-156567 | Yersinia ruckeri YR71 |

*^a^* Positions obtained by blastn (megablast) analysis.

*^b^* pYT3 lacks the IncA/C-specific *repA* replication initiation gene (see Figure S1).
