# Supplementary material for: The Master Activator of IncA/C Conjugative Plasmids Stimulates Genomic Islands and Multidrug Resistance Dissemination
Source: PLoS Genet. 2014 Oct 23;10(10):e1004714. doi: 10.1371/journal.pgen.1004714 (PMC4207636; doi:10.1371/journal.pgen.1004714)
Supplement: Table S3 — AcaCD-regulated promoters identified by ChIP-exo and 5′-RACE. (DOCX) [file pgen.1004714.s007.docx]

**Table S3.** AcaCD-regulated promoters identified by ChIP-exo and 5’-RACE.

| **MACS ChIP peaks calls** | | **AcaCD Motifs** | | | | **AcaCD regulated promoters** | | | | | |
| --- | --- | --- | --- | --- | --- | --- | --- | --- | --- | --- | --- |
| **Summit position** | **Peak Height** | **Start** | **End** | **Orientation** | ***p*-value** | **Transcription start site position** | **5'-RACE signal height***^a^* | **Oriented downstream gene** | **pVCR94Δ*acaCD* gene expression** | **pVCR94Δ*acaCD* p*acaDC*^3xFLAG^ induced gene expression** | **Predicted operon** |
| 6505 | 5567 | 6466 | 6494 | Positive | 2.7E-09 | 6529-6531 | 812 | *vcrx012* | 33 | 132 | *vcrx008-vcrx026* |
| 20410 | 3306 | 20377 | 20405 | Negative | 2.5E-08 | - | - | *vcrx035* | 0 | 0 | *-* |
|  |  | 20382 | 20410 | Positive | 5.3E-10 | 20445-20447 | 34 | *vcrx036* | 0 | 320 | *vcrx036-vcrx044* |
| 35025 | 8308 | 35010 | 35038 | Negative | 3.6E-07 | 34973-34975 | 3458 | *vcrx059* | 0 | 297 | *vcrx059-vcrx056* |
|  |  | 35015 | 35043 | Positive | 9.9E-10 | 35078-35080 | 853 | *traI* | 9 | 964 | *traI(vcrx060)-vcrx069* |
| 42805 | 1775 | 42725 | 42753 | Positive | 6.6E-11 | 42789-42791 | 473 | *vcrx068* | 0 | 550 |  |
| 43435 | 3860 | 43391 | 43419 | Negative | 5.6E-07 | - | - | *-* | - | - | *-* |
|  |  | 43396 | 43424 | Positive | 3.5E-09 | 43460-43462 | 5551 | *traL* | 0 | 218 | *traL(vcrx070)-traA(vcrx075)* |
| 46665 | 19463 | 46614 | 46642 | Positive | 6.2E-10 | 46677-46679 | 2284 | *traV* | 4 | 2750 |  |
| 47745 | 6315 | 47669 | 47697 | Negative | 3.2E-06 | - | - | *-* | - | - | *-* |
|  |  | 47674 | 47702 | Positive | 1.7E-11 | 47738-47740 | 914 | *vcrx076* | 3 | 2495 | *-* |
| 53415 | 4862 | 53355 | 53383 | Negative | 5.7E-10 | - | - | *-* | - | - | *-* |
|  |  | 53360 | 53388 | Positive | 5.6E-07 | 53424-53426 | 3673 | *dsbC* | 0 | 653 | *dsbC(vcrx077)-traU(vcrx083)* |
| 60445 | 9401 | 60398 | 60426 | Positive | 6.6E-11 | 60461-60463 | 2427 | *traN* | 282 | 2120 | *-* |
| 65015 | 19009 | 65014 | 65042 | Negative | 4.3E-07 | 64976-64978 | 423 | *vcrx086* | 17 | 1093 | *-* |
|  |  | 65019 | 65047 | Positive | 2.4E-13 | 65081-65083 | 2875 | *vcrx087* | 5 | 3445 | *-* |
| 75305 | 5602 | 75267 | 75295 | Positive | 2.3E-10 | - | - | *vcrx098* | 3 | 8357 | *vcrx098-vcrx103* |
| 86685 | 6039 | 86650 | 86678 | Positive | 3.0E-12 | 86713-86715 | 720 | *vcrx114* | 1 | 2931 | *vcrx114-vcrx119* |
| 94630 | 1772 | 94587 | 94615 | Positive | 2.5E-09 | 94651-94652 | 302 | *vcrx128* | 0 | 349 | *vcrx128-vcrx129* |
| 106830 | 2224 | 106760 | 106788 | Negative | 6.1E-06 | - | - | *-* | - | - | *-* |
|  |  | 106765 | 106793 | Positive | 7.3E-10 | 106827-106829 | 133 | *vcrx140* | 14 | 522 | *vcrx139-vcrx141* |
| 109120 | 2437 | 109039 | 109067 | Negative | 1.8E-09 | - | - | *-* | - | - | *-* |
| 118045 | 3968 | 109044 | 109072 | Positive | 1.0E-11 | 109108-109110 | 1315 | *traF* | 68 | 2187 | *traF(vcrx142)-traG(vcrx144)* |
| 120151 | 5519 | 120163 | 120191 | Negative | 2.1E-10 | 120126-120128 | 120 | *vcrx152* | 149 | 1480 | *vcrx152-vcrx151* |

*^a^* 5'-RACE signal height of pVCR94Δ*acaCD* p*acaDC*^3xFLAG^ induced.
