# Supplementary material for: The Master Activator of IncA/C Conjugative Plasmids Stimulates Genomic Islands and Multidrug Resistance Dissemination
Source: PLoS Genet. 2014 Oct 23;10(10):e1004714. doi: 10.1371/journal.pgen.1004714 (PMC4207636; doi:10.1371/journal.pgen.1004714)
Supplement: Table S4 — Primers used in this study. (DOCX) [file pgen.1004714.s008.docx]

**Table S4.** Primers used in this study.

| **Name** | **Nucleotide sequence (5’ to 3’)***^a,b,c^* |
| --- | --- |
| AcaDF-NdeI | CATATGTTGGAACTGGATATTATTGGTGCGT |
| AcaCR-HindIII | AAGCTTAGCACACTCTTTCTCCCCACCA |
| setDF-NdeI | CATATGACAACCAATCAACAAGACTTCTAT |
| setCR-HindIII | AAGCTTGTTCTCAAAAAGTGCTCTAGTG |
| FlagF | ACGAATAAGCTTGACTACAAAGACCATGACGGTGATTA |
| FlagR | GAATAAGCTTGTCGACCTACTTGTCATCGTCATCCTTGTA |
| 94DelXnoFRT.for | GGGTAGAATAAGCCTCGATATAGTCATGTGACTAAAAGGCGGAATAGGAACTTCAAGAAT |
| 94DelXnoFRT.rev | TCGTTAACTGCACATTCGGGATATTTCTCTATATTCGCGGAGAGCGCTTTTGAAGCTCA |
| 94Delvcrx025.for | CAGGTGTAAACAGTAACCAAGAAAACAAACAGGAATAACGGTGTAGGCTGGAGCTGCTTC |
| 94Delvcrx025.rev | ATGTAAACCTTATTCATGTGTCACATGATTAAGATCTATTACATATGAATATCCTCCTTA |
| 94Delvcrx146.for | TTTTGTATGGCTAACAACGCAGTTTAAAGGGTGGCAAACAGTGTAGGCTGGAGCTGCTTC |
| 94Delvcrx146.rev | GGAGAGCCGCAGACAGAACTAGCGCCTTGGCTTTGTTCTTCATATGAATATCCTCCTTA |
| 94Delvcrx150.for | AAGATGAGCGCCCAAACTTTGCGGCGCTCATTTTTTTATTACATATGAATATCCTCCTTA |
| 94Delvcrx150.rev | CGTGATTCACTTAAAGGAAAACTGATGGGCTCCGTATGACGTGTAGGCTGGAGCTGCTTC |
| 94DelacaD.for | GCTATCTATCGCAACCTTCGTGATTTGTGAGGGGGGCGGAGTGTAGGCTGGAGCTGCTTC |
| 94DelacaD.rev | CTGTAACCCAGCGACCCAATGTACCAGAGTTGCCAATGTTCATATGAATATCCTCCTTA |
| 94DelacaC.for | ACTGCAACAGTCTCTACAGGGACGTGGGAGGTGGAGATAAGTGTAGGCTGGAGCTGCTTC |
| 94DelacaC.rev | GCGCTCATCTTCTGGTCCGAAATGTCATAGTCTACTCATTACATATGAATATCCTCCTTA |
| vcrx148(acaD).for | NNNNNNGAATTCAAGGAGGAATAATAAATGTTGGAACTGGATATTATT |
| vcrx148(acaD).rev | NNNNNNGAATTCTCATCTCCACCTCCCACGT |
| vcrx149(acaC).for | NNNNNNGAATTCAAGGAGGAATAATAAATGAACATTGGCAACTCTGGTA |
| vcrx149(acaC).rev | NNNNNNGAATTCTCAAGCACACTCTTTCTCCCCA |
| vcrx146.for | NNNNNNGAATTCAAGGAGGAATAATAAATGGATAAATGCCAATTGATA |
| vcrx146.rev | NNNNNNGAATTCTCAGCTTGCCTCCTTATGT |
| vcrx150.for | NNNNNNGAATTCAAGGAGGAATAATAAATGAAAGACCACGAGGAGTTCTCTA |
| vcrx150.for | NNNNNNGAATTCTTACTTCTTCTGTGGGATA |
| Op-lacZ-F | TGGCCTTTTTGCGTGGCCAGTGCCAAGCTTGCATGCTGCAGATTCGACTCGAGCAGGAAACAGCTATGACCA |
| Op-lacZ-R | TCAAACATGAGAATTAATTCCGGGGATCCGTCGACATGCATTTATTTTTGACACCAGACCAA |
| promtraFpstI.for | NNNNNNCTGCAGGTTTGGTCAATTCTTTCACAT |
| promtraFpstI.rev | NNNNNNCTGCAGCCATGCTCGATATGGTAGA |
| promtraIpstI.for | NNNNNNCTGCAGCATCAAAAATTGTCGATGA |
| promtraIpstI.rev | NNNNNNCTGCAGCTATCGTATTTCTCGTCGCTA |
| promtraLpstI.for | NNNNNNCTGCAGCCGATCCAGTAAACGCAA |
| promtraLpstI.rev | NNNNNNCTGCAGTTCTTTTCCTAGTGGCCTGTA |
| promvcrx146pstI.for | NNNNNNCTGCAGTACTCTTTACCTCCAGTTTACCA |
| promvcrx146pstI.rev | NNNNNNCTGCAGTTAAACTGCGTTGTTAGCCAT |
| promvcrx152pstI.rev | NNNNNNCTGCAGCAAAGATTGCTTTTAGATTGCTT |
| promvcrx152pstI.rev | NNNNNNCTGCAGCAATTCCTATCCAATTCCTA |
| RepA144.F | ATGGACCACCAGCTAGAAAGTA |
| RepA144.R | GGGTTGAACTGCACATAGCA |
| SGI-1attL.for | GAGCAGGGCAAAGCGCAGCTA |
| SGI-1attL.rev | GGTTCGTAGTCGCGCTTT |
| SGI-1attR.for | GCTCGAAGAGGTAGAGCAGTA |
| SGI-1attR.rev | CGATTTGTTGTCAAAGCGGTA |
| EcU7-L12.for | ACATCTACAACAGGGCAAAG |
| Ec104D.rev | AACCATTTTGAGGTCACACA |
| GIVmi-A | ATCAGATGTTGCCGAAGAGC |
| GIVmi-B | GTACAAGCCATTACCATCTGC |
| GIVmi-C | GTAACGTGATACAGTTACTC |
| GIVmi-D | AGGATCAGGCTTAAACCAA |
| GIVmi-res2F | CTAGCAGAATTCCTTGATGAGGCGCATCGTGG |
| GIVmi-res2R | CACTGAGGATCCTTATCTACGAGCTAACGGTGTTC |
| VMD_06510.qvmi.F1 | GGGCAAGCTCAACGCACGA |
| VMD_06510.qvmi.R1 | ACGTGCGTCAGCCAGGGAT |
| VMD_06490.qvmi.F2 | AGCTCGAACGATAACGGCTGC |
| VMD_06490.qvmi.R2 | TGCCAGGTAGATTTGGTTGTCGC |
| VMD_06480.F1 | TGGGTTTCAACGGCAATGGCA |
| VMD_06480.R1 | ACCGATTCTGGTTTGTCTGTAACAACT |
| VMD_06410.F1 | GCTGCGCACCCTGATCACAT |
| VMD_06410.R1 | CGGGATCTGCTTGGCATGGG |
| gyrA.qvmich.F2 | CCCGTGGTTGGGATCTTGGC |
| gyrA.qvmich.R2 | CGCTTGCGCTTGTTGCTCAG |
| rpoZ.qvmich.F1 | CCGCGCTCGTCAAATGCAATC |
| rpoZ.qvmich.R1 | GCCAGCTCTGCCGCTTCTTG |
| TruSeq-B-dT24-VN | AGACGTGTGCTCTTCCGATCTTTTTTTTTTTTTTTTTTTTTTTTVN |
| IGA-A0-up | /5AmMC6/ACACTCTTTCCCTACACGACGCTCTTCCGATCT |
| IGA-A0-down-T/A | GATCGGAAGAGCGTCGTGTAGGGAAAGAGTGTAGAT |
| IGA-PCR-PE-F | AATGATACGGCGACCACCGAGATCTACACTCTTTCCCTACACGACGCTCTTCCGATCT |
| TrueSeq-MPEX-R | CAAGCAGAAGACGGCATACGAGAT-index-GTGACTGGAGTTCAGACGTGTGCTCTTCCGATC |
| TrueSeq-3'-hybrid-B0 | /5Phos/rArGrArUrCrGrGrArArGAGCACACGTCT/3AmMO/ |
| 5'-hybrid-A0 | ACACGACGrCrUrCrUrUrCrCrGrArUrCrU |
| DSN-TrueSeq-F | CACGACGCTCTTCCGATCT |
| DSN-TrueSeq-R | AGACGTGTGCTCTTCCGATCT |

*^a^* Restriction sites are underlined and -index- corresponds to a barcode, i.e. a specific combination of 6 bp for each sample (see Table S5).

*^b^* Mixed bases: r: A or G; V: A, C or G; N: A, T, C or G

*^c^* Modifications: /5AmMC6/ : 5' amino modifier C6; /5Phos/ : 5’ phosphorylation; /3AmMO/ : 3' amino modifier
