# Supplementary material for: The Master Activator of IncA/C Conjugative Plasmids Stimulates Genomic Islands and Multidrug Resistance Dissemination
Source: PLoS Genet. 2014 Oct 23;10(10):e1004714. doi: 10.1371/journal.pgen.1004714 (PMC4207636; doi:10.1371/journal.pgen.1004714)
Supplement: Table S5 — Illumina libraries sequenced in this study. (DOCX) [file pgen.1004714.s009.docx]

**Table S5.** Illumina libraries sequenced in this study.

| **Sequencing line** | **Library type** | **Experimental condition** | **Replicate** | **Index in TrueSeq-MPEX-R oligo** | **Index read by instrument** | **Sequenced paired-end reads (millions)** |
| --- | --- | --- | --- | --- | --- | --- |
| 1 | ChIP-exo | pVCR94Δ*acaCD*  p*acaDC*^3xFLAG^ induced | 1 | AGGCTA | TAGCCT | 3.7 |
|  |  |  | 2 | GCTATA | TATAGC | 3.5 |
|  |  |  | 3 | TAGATA | TATCTA | 3.4 |
|  | Total RNA-seq | pVCR94 wild-type | 1 | AGCCGC | GCGGCT | 3.9 |
|  |  |  | 2 | CGGTTG | CAACCG | 3.8 |
|  |  |  | 3 | GTAATT | AATTAC | 3.0 |
|  |  | pVCR94Δ*acaCD* | 1 | TGACCG | CGGTCA | 2.6 |
|  |  |  | 2 | CCGCTC | GAGCGG | 3.2 |
|  |  |  | 3 | GAGGTC | GACCTC | 2.5 |
|  |  | pVCR94Δ*acaCD*  p*acaDC*^3xFLAG^ induced | 1 | AGTTGG | CCAACT | 4.9 |
|  |  |  | 2 | TCGTCC | GGACGA | 3.9 |
|  |  |  | 3 | TTGCGC | GCGCAA | 3.7 |
| 2 | Total RNA-seq | pVCR94 wild-type | 4 | TAAGCC | GGCTTA | 6.2 |
|  |  |  | 5 | ATTCCG | CGGAAT | 6.8 |
|  |  |  | 6 | ACGCGG | CCGCGT | 5.8 |
|  |  | pVCR94Δ*acaCD* | 4 | GACTCC | GGAGTC | 6.2 |
|  |  |  | 5 | CGATCC | GGATCG | 4.1 |
|  |  |  | 6 | AACCTG | CAGGTT | 6.8 |
|  |  | pVCR94Δ*acaCD*  p*acaDC*^3xFLAG^ induced | 4 | GAGAGT | ACTCTC | 5.6 |
|  |  |  | 5 | AGTTGG | CCAACT | 4.9 |
|  |  |  | 6 | TCGTCC | GGACGA | 5.0 |
|  | 5'-RACE | pVCR94Δ*acaCD* | 1 | CAGCAG | CTGCTG | 5,7 |
|  |  | pVCR94Δ*acaCD*  p*acaDC*^3xFLAG^ induced | 1 | CTAACG | CGTTAG | 6.0 |
