# Supplementary material for: The Master Activator of IncA/C Conjugative Plasmids Stimulates Genomic Islands and Multidrug Resistance Dissemination
Source: PLoS Genet. 2014 Oct 23;10(10):e1004714. doi: 10.1371/journal.pgen.1004714 (PMC4207636; doi:10.1371/journal.pgen.1004714)
Supplement: Text S1 — Additional experimental procedures. (DOCX) [file pgen.1004714.s011.docx]

# Text S1. Additional experimental procedures

## AcaCD ChIP-exo experiments

ChIP-exo experiments were performed in biological triplicates, each using 10 ml of *E. coli* MG1655 Rf bearing pVCR94Δ*acaCD* and p*acaDC*^3xFLAG^ grown to an OD_600nm_ of ~0.3 with agitation in an orbital shaker at 37°C. Cultures were then induced with 100 μM IPTG and incubated for one hour at 37°C. Induced cultures were next subjected to 1% formaldehyde crosslinking for 20 min at 25°C followed by quenching with 0.125 M glycine. Cells were washed twice with ice-cold TBS 1X (20 mM Tris-HCl (pH 7.5), 150 mM NaCl) and resuspended in 300 μl of lysis buffer (50 mM HEPES-KOH (pH 7.5), 140 mM NaCl, 1 mM EDTA (pH 7.5), 1% (v/v) Triton X-100, 0.1 % (v/v) Na-deoxycholate, 1 mM PMSF, 2 μM Pepstatin, 0.72 μM Leupeptin, 0.26 μM Aprotinin). Cells were lysed and their DNA was simultaneously sheared by sonication to an average fragment size of 200-400 bp using a Bioruptor UCD-200 instrument (Diagenode) at high power setting for 35 cycles, each consisting of 30 sec on and 30 sec off inside a 4°C temperature-controlled water bath. Lysates were then clarified by centrifugation for 10 min at 16,000 g in a 4°C pre-cooled microcentrifuge and pellets were discarded. The recovered supernatant for each tube was adjusted to 800 μl with lysis buffer, and 1 μl of 3.8 μg/μl Anti-FLAG M2 antibody (Sigma) was added for an overnight incubation on a rotator at 4°C. Immunoprecipitation was performed using 50 μl of Protein A magnetic Dynabeads (Life Technologies), pre-blocked with BSA blocking solution (5 mg/ml BSA, 1X PBS) for 4 hours at 4°C on a rotator. Magnetic dynabeads were then washed twice, respectively with lysis buffer, high salt lysis buffer (500 mM NaCl instead of 140 mM NaCl in lysis buffer), ChIP wash buffer (10 mM Tris-HCl (pH 8.0), 250 mM LiCl, 0.5 % (v/v) NP40, 0.5 % (v/v) Na-deoxycholate, 1 mM EDTA (pH 8.0)), and once with TE 1X (10 mM Tris-HCl (pH 8.0), 1 mM EDTA (pH 8.0)). Magnetic dynabeads were then resuspended in ChIP-exo buffer (1X NEBuffer 4 [New England BioLabs], 1 mM PMSF, 2 μM Pepstatin, 0.72 μM Leupeptin, 0.26 μM Aprotinin) with 40 U of T7 Exonuclease (New England BioLabs), 60 U of RecJf (New England BioLabs), and 20 μg of RNAse A (Roche) and incubated at 37°C with shaking in a Multi-Therm thermomixer (Benchmark Scientific) for 60 min at 1100 rpm. The dynabeads were washed again as described above and 50 μl of ChIP elution buffer (50 mM Tris-HCl (pH 8.0), 10 mM EDTA (pH 8.0), 1% (w/v) SDS) was added. Magnetic dynabeads were incubated at 65°C with agitation for 10 min and supernatant was recovered. Crosslinks were reversed by adding 180 μl of TE/SDS (10 mM Tris-HCl (pH 8.0), 1 mM EDTA (pH 8.0), 1% (w/v) SDS) and incubating the reaction at 65°C overnight. 20 μg of RNAse A (Roche) was added and reactions were incubated at 37°C for 30 min, which was followed by the addition of 9.2 U of Proteinase K (New England BioLabs) and an additional incubation of 2 hours at 37°C. DNA was finally purified using the ChIP DNA Clean and Concentrator kit (Zymo Research) according to the manufacturer's specifications, except that 7 volumes of ChIP DNA Binding Buffer were added to each sample instead of 5 volumes mentioned in the provided protocol. Purified DNA was then quantified using the Quant-it PicoGreen dsDNA assay kit (Life Technologies) according to the manufacturer's specifications using a Synergy HT plate reader equipped with a Take3 Multi-Volume Plate (BioTek).

## ChIP-exo libraries preparation

ChIP-exo libraries were generated using 5 ng of purified DNA that was first dA-tailed using 5 U of Terminal Transferase in 1X Terminal Transferase Reaction Buffer (New England BioLabs) supplemented with 0.25 mM CoCl_2_ and 10 μM dATP for 30 min at 37°C. DNA was purified using Agencourt AMPure XP SPRI beads (Beckman Coulter) with a 1.8 ratio (beads solution volume respective to terminal transferase reaction volume) as described elsewhere [[1](#_ENREF_1)]. Next, 5' ends of DNA were phosphorylated using 10 U of T4 Polynucleotide Kinase in 1X T4 Polynucleotide Kinase Reaction Buffer (Enzymatics) with 1 mM ATP at 37°C for 30 min. DNA was purified again with Agencourt AMPure XP SPRI beads (Beckman Coulter) with the same ratio of 1.8. Then, the second strand of dA-tailed DNA molecules was synthesized using 5 U of *Bst* DNA polymerase full length in 1X ThermoPol Reaction Buffer (New England BioLabs) with 1 mM dNTPs and 0.5 μM of the TrueSeq-B-dT24-VN oligo (see Table S4). Reaction was then incubated from 40°C to 70°C for 1 hour (temperature increment of 0.5°C per min). DNA was purified with Agencourt AMPure XP SPRI beads (Beckman Coulter) with a 1.0 ratio. The 3'-T-IGA-A0 adaptor (IGA-A0-up annealed with IGA-A0-down-T/A, see Table S4; 30 nM adaptor final concentration) was then ligated using 1,200 U of T4 Rapid DNA ligase in 1X Quick Ligation Reaction Buffer (Enzymatics) at room temperature for 15 min. Then, PEG concentration was reduced by adding a volume of water equal to half of the reaction volume directly to the reaction tube and DNA was purified with Agencourt AMPure XP SPRI beads (Beckman Coulter) with a 0.8 ratio to eliminate unligated adaptors. Nicks were repaired using 5 U of Taq-B DNA polymerase in 1X Taq-B Reaction Buffer (Enzymatics) with 0.125 mM dNTPs at 66°C for 20 min. DNA was purified once again with Agencourt AMPure XP SPRI beads (Beckman Coulter) with a 1.8 ratio. DNA molecules were amplified by real-time PCR using 0.5 U of VeraSeq 2.0 DNA polymerase in 1X VeraSeq 2.0 DNA polymerase Reaction Buffer (Enzymatics) with 0.25X SYBR green I, 0.2 mM dNTPs, and 0.4 μM of IGA-PCR-PE-F and TrueSeq-MPEX-R oligos (see Table S4) with a specific index for each sample (see Table S5). The PCR conditions were as follows: (i) 30 sec at 98°C; (ii) ~15-20 cycles (step transferred to final elongation before reaching an amplification plateau) of 15 sec at 98°C, 15 sec at 60°C, and 15 sec at 72°C; and (iii) 2 min at 72˚C. The resulting libraries were purified with Agencourt AMPure XP SPRI beads (Beckman Coulter) with a ratio of 1.0, before evaluating the library size and concentration using a 2100 Bioanalyzer instrument (Agilent Technologies). Indexed libraries were pooled together in equal quantity to obtain approximately the same amount of reads per sample (see Table S5).

## Total RNA extractions

Total RNA extractions were done with three biological replicates. 5 ml of *E. coli* MG1655 Rf pVCR94Δ*acaCD* p*acaDC*^3xFLAG^ was grown to an OD_600nm_ of ~0.3 as described for ChIP-exo experiments. The culture was then induced with 100 μM IPTG (pVCR94Δ*acaCD* p*acaDC*^3xFLAG^ induced) for one hour and reached a final OD_600nm_ of ~0.6. 5 ml of *E. coli* MG1655 Rf pVCR94 wild-type and *E. coli* MG1655 Rf pVCR94Δ*acaCD* were also grown to an OD_600nm_ of ~0.6 for RNA extraction. Total RNA was then extracted for all cultures using the Direct-zol RNA MiniPrep kit (Zymo Research) according to the manufacturer's specifications. Purified total RNA was treated with 1.5 U of DNAse I in 1X DNAse I Reaction Buffer (Zymo Research) at 37°C for 20 min. DNAse-treated RNA samples were then purified using the RNA Clean & Concentrator-5 kit (Zymo Research) according to the manufacturer's specifications. The quality and concentration of total RNA extractions were evaluated using a 2100 Bioanalyzer instrument (Agilent Technologies).

## Total RNA-seq libraries preparation

Total RNA-seq libraries were constructed in biological triplicates, each divided in two technical replicates, for a total of six libraries per experimental condition. Purified total RNA samples were fragmented at 95°C for 7 min in RNA fragmentation buffer (40 mM Tris-acetate (pH 8.1), 125 mM KOAc, 37.5 mM MgOAc). Fragmented RNA was next purified using the RNA Clean & Concentrator-5 kit (Zymo Research) according to the manufacturer's specifications. 5'-end of RNA molecules were then dephosphorylated using 2.5 U of Antarctic Phosphatase in 1X Antarctic Phosphatase Reaction Buffer (New England BioLabs) with 20 U of RNAse Inhibitor (Enzymatics) at 37°C for 30 min. RNA was purified again using the RNA Clean & Concentrator-5 kit (Zymo Research) according to the manufacturer's specifications. The TrueSeq-3'-hybrid-B0 oligo (see Table S4; 2 μM final concentration) was then ligated using 30 U of T4 RNA ligase 1 in T4 RNA Ligase 1 Reaction Buffer (Enzymatics) with 10% DMSO and 20 U of RNAse Inhibitor (Enzymatics) at 37°C for 60 min. RNA was purified with Agencourt RNAclean XP SPRI beads (Beckman Coulter) with a 1.8 ratio. 5' end of RNA molecules was then phosphorylated using 10 U of T4 Polynucleotide Kinase in 1X T4 Polynucleotide Kinase Reaction Buffer (Enzymatics) containing 1 mM ATP and 40 U of RNAse Inhibitor (Enzymatics) for 30 min at 37°C. RNA was purified again with Agencourt RNAclean XP SPRI beads (Beckman Coulter) with a 1.8 ratio. The 5'-hybrid-A0 oligo (see Table S4; 2 μM final concentration) was then ligated using the same conditions as for 3'-hybrid-0 oligo ligation. RNA was then purified with Agencourt RNAclean XP SPRI beads (Beckman Coulter) with a 1.8 ratio. RNA was reverse transcribed to cDNA using 200 U of M-MuLV Reverse Transcriptase in 1X MuLV Reaction Buffer (Enzymatics) containing 1 mM dNTPs, 40 U of RNAse Inhibitor (Enzymatics), and 0.5 μM of the DSN-TrueSeq-R oligo (see Table S4) using the following conditions: 42°C for 10 min, 42°C to 50°C for 40 min (temperature increment of 0.2°C per min), and 50°C for 10 min. cDNAs were purified with Agencourt AMPure XP SPRI beads (Beckman Coulter) with a ratio of 1.8. cDNA molecules were then amplified by real-time PCR using 0.5 U of VeraSeq 2.0 DNA polymerase in 1X VeraSeq 2.0 DNA polymerase Reaction Buffer (Enzymatics) with 0.25X SYBR green I, 0.2 mM dNTPs, and 0.4 μM of DSN-TrueSeq-F and DSN-TrueSeq-R oligos (see Table S4). The PCR conditions were as follows: (i) 30 sec at 98°C; (ii) ~15-20 cycles (step transferred to final elongation before reaching an amplification plateau) of 15 sec at 98°C, 15 sec at 55°C, and 15 sec at 72°C; and (iii) 2 min at 72˚C. Amplified DNA molecules were purified with Agencourt AMPure XP SPRI beads (Beckman Coulter) with a ratio of 1.0. DNA molecules corresponding to the rRNA transcripts were depleted using the Duplex-specific nuclease as described elsewhere (Evrogen; [[2](#_ENREF_2)]). Total RNA-seq libraries were purified with Agencourt AMPure XP SPRI beads (Beckman Coulter) with a ratio of 1.6. Libraries were amplified again by real-time PCR using the same conditions as for ChIP-exo libraries amplification (see ChIP-exo libraries preparation section). The resulting libraries were purified with Agencourt AMPure XP SPRI beads (Beckman Coulter) with a ratio of 0.9, before evaluating the library size and concentration using a 2100 Bioanalyzer instrument (Agilent Technologies). Indexed libraries were pooled together in equal quantity to obtain approximately the same amount of reads per sample (see Table S5).

## Genome-wide 5'-RACE RNA libraries preparation

Equal quantities of purified total RNA from either pVCR94Δ*acaCD* p*acaDC*^3xFLAG^ induced or pVCR94Δ*acaCD* replicates were pooled to obtain a final amount of 2 μg. Pooled total RNA was fragmented at 75°C for 10 min in RNA fragmentation buffer (40 mM Tris-acetate (pH 8.1), 125 mM KOAc, 37.5 mM MgOAc). Fragmented RNA was purified using the RNA Clean & Concentrator-5 kit (Zymo Research) according to the manufacturer's specifications. The TrueSeq-3'-hybrid-B0 oligo (see Table S4) was then ligated as described in the total RNA-seq libraries preparation section. 3'-adaptor ligated RNA was purified using the RNA Clean & Concentrator-5 kit (Zymo Research) according to the manufacturer's specifications. RNA was then treated with 3 U of XRN-1 (New England BioLabs) in 1X NEBuffer 3 containing 20 U of RNAse Inhibitor (Enzymatics) at 37°C for 60 min. Treated RNA was purified using the RNA Clean & Concentrator-5 kit (Zymo Research) according to the manufacturer's specifications. Pyrophosphate from the 5' end of triphosphate RNA was removed using 5 U of RppH (New England BioLabs) in 1X NEBuffer 2 containing 40 U of RNAse Inhibitor (Enzymatics) at 37°C for 30 min. RNA was purified again using the RNA Clean & Concentrator-5 kit (Zymo Research) according to the manufacturer's specifications. The 5'-hybrid-A0 oligo (see Table S4) was then ligated as described in the total RNA-seq libraries preparation section. RNA was then purified with Agencourt RNAclean XP SPRI beads (Beckman Coulter) with a 1.8 ratio. RNA was reverse transcribed to cDNA as described in the total RNA-seq libraries preparation section. cDNAs were purified with Agencourt AMPure XP SPRI beads (Beckman Coulter) with a ratio of 1.8. Libraries were finally amplified by real-time PCR using the same conditions as for ChIP-exo libraries amplification (see ChIP-exo libraries preparation section). The resulting libraries were purified with Agencourt AMPure XP SPRI beads (Beckman Coulter) with a ratio of 0.9, before evaluating the library size and concentration using a 2100 Bioanalyzer instrument (Agilent Technologies).

## Illumina sequencing

Illumina sequencing was performed on a Illumina HiSeq 2000 Sequencing system at the Plateau de Biologie Moléculaire et génomique fonctionnelle of the Institut de Recherche Cliniques de Montréal. Approximately 3 to 6 million paired-end reads of 50 bp were obtained for each prepared libraries. Sample multiplexing relied on a 6-bp index located within the TrueSeq-MPEX-R oligo (see Table S4 and S5). Samples were pooled in two separated mixes and sequenced on two separated lines (Table S5).

## ChIP-exo data analysis

Illumina reads corresponding to the AcaCD protein complex boundaries (forward read of mate pair) of triplicate ChIP-exo prepared libraries (see Table S5) were aligned using the BWA program [[3](#_ENREF_3)] on the *E. coli* MG1655 genome (GenBank U00096.3) as well as pVCR94ΔX (GenBank NC_023291). Alignment triplicates (Pearson's correlation = 0.97) were filtered by SAMtools view [[4](#_ENREF_4)] to discard aligned reads with a quality score below 10. Filtered alignment triplicates were then combined using SAMtools merge and sorted according to the pVCR94ΔX DNA sequence using SAMtools sort. Normalized read density (Figure 3B) as well as enrichment peaks were calculated by MACS [[5](#_ENREF_5)] using the following options: -f BAM, -g 4700000 -S, -w, -p 1e-30, --nomodel, --nolambda, --keep-dup 100 --call-subpeaks. Subpeaks (referred as “peaks” for the rest of the paper; Figure 3B and Table S3) with a summit height below 25X of the theoretical bp coverage (obtained by calculating the ratio between the number of reads used by MACS and the combined *E. coli* MG1655 and pVCR94ΔX genome size) were discarded to eliminate the background signal. One enrichment peak with a summit height above the background signal was identified manually due to a MACS false negative result and indicated with an asterisk in Figure 3B. AcaCD-binding logo was found by the MEME tool [[6](#_ENREF_6)] using nucleotide sequences corresponding to MACS pVCR94ΔX peaks summits ± 100 bp with the any number of repetitions option and a maximum motif length of 30 bp (Figure 3C). Precise locations of AcaCD-binding motifs were searched using MAST [[7](#_ENREF_7)] against the complete pVCR94ΔX sequence with the AcaCD MEME logo, and intersected with MACS peaks. The MAST option to treat the negative DNA strand as a separated sequence was used, and only motifs with *p*-values below 1.0e-05 positioned within pVCR94ΔX MACS peaks were considered as significant hits (Figure 3B and Table S3). AcaCD-binding motifs inside SGI1 and MGI*Vmi*573 were also calculated by MAST using the same method, except that significant peaks were not selected for their localization within a MACS peak (Figure 4A and Table 1). To obtain a near single nucleotide resolution with our ChIP-exo data, we next treated the filtered and sorted BWA's sequences mapped on pVCR94ΔX to conserve only the first nucleotide of each forward read. Finally, 1 bp resolution ChIP-exo reads density (Figure 3D, E), as well as full reads ChIP-exo density, were calculated with Bedtools’ genomecov function for separated DNA strands.

## Total RNA-seq data analysis

Illumina reads obtained from total RNA-seq libraries (see Table S5) were trimmed using Trimmomatic tool [[8](#_ENREF_8)] to discard nucleotides with a quality score below 30. Trimmed reads with a length below 36 bp or with no corresponding mate pair read were also discarded. Trimmed reads were then aligned on the *E. coli* MG1655 genome (GenBank U00096.3) as well as pVCR94ΔX (GenBank NC_023291) by the Rockhopper software, specialized for analyzing bacterial RNA-seq data, with default parameters [[9](#_ENREF_9)]. RPKM and expression values of pVCR94ΔX genes were calculated by the software as well as differential gene expression between RNA-seq experimental conditions (see Dataset S1 and Table S3). Statistically, genes between two given conditions were considered differentially expressed by Rockhopper if the calculated *q*-value was equal of below 0.01 (see Dataset S1). Pearson's correlations between conditions (pVCR94ΔX wild-type, pVCR94Δ*acaCD,* and pVCR94Δ*acaCD* p*acaDC*^3xFLAG^ induced) were calculated from Rockhopper's calculated genes expression values. Based on the proximity of ORFs and the RNA-seq data analysis, operons were also predicted by Rockhopper (see Dataset S1 and Table S3). To generate RNA-seq density files, trimmed reads were also aligned by BWA on the *E. coli* MG1655 genome (GenBank U00096.3) as well as pVCR94ΔX (GenBank NC_023291). Alignment replicates (see Table S5) were filtered by SAMtools view to discard aligned reads with a quality score below 10 and combined using SAMtools merge. Pooled as well as individual filtered alignment replicates were then sorted according to the pVCR94ΔX DNA sequence with SAMtools sort. RNA-seq reads density for individual and pooled replicates were finally calculated with Bedtools genomecov for separated DNA strands (Figure 3B).

## Genome-wide 5'-RACE data analysis and AcaCD-regulated promoters characterization

Illumina reads corresponding to the start of transcribed RNAs (forward read of mate pair) obtained from the 5'-RACE libraries (see Table S5) were trimmed, aligned by BWA, filtered and sorted by SAMtools as described in the previous sections. Filtered and sorted sequences were then treated to conserve only the first nucleotide of each read, corresponding to potential transcription start sites. 1 bp resolution 5'-RACE reads density was calculated with Bedtools genomecov for separated DNA strands (Figure 3B, D, E). The localization of AcaCD-binding motifs in promoters was next analyzed for all transcription start sites located between an AcaCD-binding motif and a gene in the same orientation (Figure 3B, D, Figure S4 and Table S3) using the Versatile Aggregate Profiler (VAP) [[10](#_ENREF_10)]. Two pairs of divergent promoters, located in the *vcrx059*-*traI* (Figure 3E) and *vcrx086*-*vcrx087* intergenic regions, were excluded from this analysis because of the presence of ChIP-exo signals from partly overlapping AcaCD motifs on the positive and negative strands. Other promoters with a transcription start site identified experimentally by 5'-RACE reported in Table S3 and Figure S4 were submitted to VAP to extract signals in a 400 bp window centered on the predicted AcaCD motif, and generate an average representation of the data.

# References

1. Rodrigue S, Materna AC, Timberlake SC, Blackburn MC, Malmstrom RR, et al. (2010) Unlocking short read sequencing for metagenomics. PLoS One 5: e11840.

2. Christodoulou DC, Gorham JM, Herman DS, Seidman JG (2011) Construction of normalized RNA-seq libraries for next-generation sequencing using the crab duplex-specific nuclease. Curr Protoc Mol Biol Chapter 4: Unit4 12.

3. Li H, Durbin R (2009) Fast and accurate short read alignment with Burrows-Wheeler transform. Bioinformatics 25: 1754-1760.

4. Li H, Handsaker B, Wysoker A, Fennell T, Ruan J, et al. (2009) The Sequence Alignment/Map format and SAMtools. Bioinformatics 25: 2078-2079.

5. Zhang Y, Liu T, Meyer CA, Eeckhoute J, Johnson DS, et al. (2008) Model-based analysis of ChIP-Seq (MACS). Genome Biol 9: R137.

6. Bailey TL, Elkan C (1994) Fitting a mixture model by expectation maximization to discover motifs in biopolymers. Proc Int Conf Intell Syst Mol Biol 2: 28-36.

7. Bailey TL, Gribskov M (1998) Combining evidence using p-values: application to sequence homology searches. Bioinformatics 14: 48-54.

8. Bolger AM, Lohse M, Usadel B (2014) Trimmomatic: a flexible trimmer for Illumina sequence data. Bioinformatics.

9. McClure R, Balasubramanian D, Sun Y, Bobrovskyy M, Sumby P, et al. (2013) Computational analysis of bacterial RNA-Seq data. Nucleic Acids Res 41: e140.

10. Coulombe C, Poitras C, Nordell-Markovits A, Brunelle M, Lavoie MA, et al. (2014) VAP: a versatile aggregate profiler for efficient genome-wide data representation and discovery. Nucleic Acids Res 42: W485-W493.
